# Renewable Feedstock Nanocarriers for Drug Delivery: Evidence Mapping and Translational Readiness

**DOI:** 10.3390/pharmaceutics18040407

**Published:** 2026-03-25

**Authors:** Renato Sonchini Gonçalves

**Affiliations:** Department of Engineering and Exact Sciences, Setor Palotina, Federal University of Paraná (UFPR), Palotina 85950-000, PR, Brazil; renato.sonchini@ufpr.br; Tel.: +55-98-985-149-235

**Keywords:** renewable resources, sustainable nanotechnology, green synthesis, bio-based nanomaterials, drug delivery systems, lignocellulosic nanomaterials, chitosan, polysaccharide nanogels, lipid nanocarriers, lifecycle assessment, quality by design, GMP scale-up

## Abstract

Sustainable nanotechnologies derived from renewable resources are increasingly being positioned at the interface of green chemistry, advanced drug delivery, and translational pharmaceutics. Over the past decade, lignocellulosic nanomaterials, chitin/chitosan platforms, polysaccharide-based nanogels and nano-enabled hydrogels, lignin- and polyphenol-derived nanostructures, and bio-based lipid nanocarriers have been engineered through progressively eco-efficient routes, including solvent-minimized self-assembly, nanoprecipitation, spray drying, hot-melt extrusion, and microfluidic-assisted fabrication. This work provides a structured evidence map of nano-enabled drug delivery and therapeutic platforms derived from renewable biological resources. Specifically, we aim to (i) identify and classify nanoplatform classes and renewable feedstocks; (ii) summarize reported pharmaceutical critical quality attributes (CQAs) and performance and safety endpoints; and (iii) appraise how “renewability” and “green” claims are evidenced (feedstock origin vs. process sustainability) and how frequently translational readiness factors (scalability, quality control, regulatory alignment) are addressed. We critically compare renewable and conventional nanomaterial platforms across key translational dimensions, including carbon footprint, batch consistency, biodegradability, functional tunability, safety/persistence, and scale-up maturity. Finally, we delineate a practical translational pathway—from biomass sourcing and fractionation to nanoformulation, characterization/stability, and GMP scale-up—highlighting cross-cutting enablers such as lifecycle assessment, EHS/toxicology risk assessment, quality-by-design, and regulatory alignment. Collectively, the evidence supports renewable nanomaterials as viable, scalable candidates for next-generation therapeutics, provided that variability control, standardized characterization, and safety-by-design principles are embedded early in development.

## 1. Introduction

The growing global demand for safer, more efficient, and environmentally responsible therapeutic technologies has placed sustainability at the center of contemporary pharmaceutical innovation [1,2]. The chemical and pharmaceutical industries remain among the sectors with the highest consumption of energy and organic solvents, contributing substantially to persistent waste, environmental toxicity, and greenhouse gas emissions [1,3]. In parallel, international organizations such as the World Health Organization, the Organisation for Economic Co-Operation and Development, and the United Nations have emphasized the urgent need to integrate the principles of green chemistry, the circular economy, and the bioeconomy into production chains, in alignment with the Sustainable Development Goals of the 2030 Agenda and the European Green Deal [1,4]. From a biomedical perspective, this scenario is intensified by the escalating incidences of chronic, infectious, and emerging diseases and by the need for more accessible and precise multimodal therapies [1]. In this context, the development of new therapeutic platforms must incorporate strategies that minimize ecotoxicological burdens, reduce petrochemical dependences, and expand the use of materials derived from renewable resources [4]. Sustainable nanotechnology emerges as an integrated response to this global landscape, offering structural and functional alternatives capable of combining advanced therapeutic performance, material safety, and environmental responsibility within a unified technological paradigm.

Despite the advances achieved over the past several decades, conventional nanotechnology continues to face structural, environmental, and pharmacotechnical limitations that restrict clinical translation and industrial feasibility [1,3,4]. Most current nanocarriers rely on synthetic polymers, high-toxicity surfactants, and fabrication processes that require volatile organic solvents, whose persistence and ecotoxicity pose significant challenges for sustainable implementation [1,3]. Furthermore, many synthetic nanomaterials exhibit limited biodegradability, prolonged systemic retention, and potential off-target interactions, complicating toxicological assessments and regulatory approval processes [1,5]. Traditional manufacturing routes often involve energy-intensive steps, high-temperature processing, and costly purification procedures, hindering scalability and limiting deployment in resource-constrained settings [1,3,4]. The absence of intrinsic bioactivity and the need for complex chemical functionalization further increase production costs and variability [1,2,4]. Collectively, these constraints underscore the urgency of adopting renewable-resource-based strategies grounded in green chemistry to overcome historical limitations and align therapeutic innovation with sustainability principles.

In this review, “nanomaterial” refers to a material presenting at least one characteristic dimension in the nanoscale (typically 1–1000 nm for drug delivery colloids, while ≤100 nm is often used in strict nanomaterial definitions), resulting in size-dependent interfacial, transport, or optical properties relevant to biomedical performance. “Nanocarrier” denotes a nanoscale delivery vehicle engineered to solubilize, protect, and deliver active pharmaceutical ingredients (APIs), encompassing droplet-based colloids (e.g., nanoemulsions), self-assembled aggregates (e.g., lipid or polymeric micelles), and discrete nanoparticles/capsules (e.g., polymeric nanoparticles, lipid nanoparticles, lipid nanocapsules, and nanogels). “Renewable nanomaterials” are nanomaterials whose building blocks are predominantly derived from renewable feedstocks (biomass), including polysaccharides (cellulose, chitin/chitosan, alginate, pectin, pullulan), lignin- and tannin-rich fractions, proteins/peptides, and plant-/microbial-derived lipids; these components may act as matrices, stabilizers, surface ligands, or structural crosslinkers. The term “nanoarchitecture” emphasizes intentionally designed nanoscale organization (e.g., core–shell nanoparticles, layer-by-layer nanocapsules, nanogels, nanofibers, and nanocomposites) that governs loading, release, and interfacial interactions. “Nanostructure” is used as a morphology-agnostic descriptor of the resulting nanoscale entity (droplet, micelle, particle, capsule, fiber, or gel nanoparticle). “Biomass-derived nanocarrier” specifies a nanocarrier whose matrix and/or stabilizing components originate from biomass, whereas “biomass-derived nanostructure” highlights the structural entity formed from such components, irrespective of whether it is used as a carrier. Throughout the manuscript, we explicitly indicate whether a reported biological effect is (i) intrinsic to the biomass-derived component exposed at the nano-interface (material-driven) or (ii) predominantly payload-mediated, with the nanostructure acting as an enabling delivery architecture.

Sustainable nanotechnology represents an evolutionary shift in nanomedicine by integrating green chemistry, bioengineering, and renewable biological inputs into the design of functional materials [1,5]. This framework promotes the replacement of petrochemical-derived reagents and toxic solvents with eco-efficient processes founded on natural feedstocks such as biopolymers, plant-derived metabolites, microbial biomass, and low-impact hybrid materials [2,4,5,6]. Beyond reducing environmental burdens, sustainable nanotechnology leverages intrinsic properties—including bioactivity, biocompatibility, biodegradability, and stimuli-responsiveness—to streamline formulations and enhance biological interactions [4,5,7]. Such materials align closely with modern regulatory expectations that prioritize clean synthesis routes, reduced carbon footprints, and circular value chains [1,5,6]. Consequently, sustainable nanotechnology consolidates itself as a strategic axis of innovation, redefining the future of drug design and delivery through environmentally aligned molecular engineering [1,2,5].

Renewable resources provide the structural foundation of sustainable nanotechnology, offering diverse biological matrices capable of generating functionally sophisticated and ecologically relevant nanosystems [2,4,8]. Lignocellulosic biomass, composed of cellulose, hemicellulose, and lignin, enables the development of nanocrystals, nanofibrils, and nanocellulose-based (nano-enabled) hydrogels with high mechanical strength, biodegradability, and excellent colloidal stability [8,9]. Metabolic plant biomass—including terpenoids, flavonoids, alkaloids, curcuminoids, quinones, and polyphenolic complexes—supplies bioactive molecules with antioxidant, antimicrobial, anti-inflammatory, and natural photosensitizing properties, enabling the construction of hybrid nanosystems with enhanced pharmacodynamic synergy [10,11]. Oleaginous biomass, represented by essential oils and plant lipids, supports the formation of nanoemulsions, micelles, and self-assembled structures capable of improving the solubility and transmembrane permeability of hydrophobic drugs [11,12]. Microbial and marine biomass—including PHA, PLA, pullulan, carrageenans, and chitosan—offers highly pure, reproducible, and biodegradable polymers compatible with low-impact synthesis routes and advanced controlled-release systems [13,14]. Together, these renewable matrices provide a versatile platform for the sustainable molecular design and engineering of next-generation therapeutic nanomaterials [2,4,13].

Sustainable synthesis routes constitute a core element of renewable nanotechnology, enabling the fabrication of advanced nanomaterials through environmentally responsible, energy-efficient, and low-toxicity processes [1,4]. Solvent-free or low-solvent techniques—including hot-melt extrusion, spray drying, mechanical milling, and supercritical fluid processing—allow the production of stable nanostructures under clean and efficient conditions [4,15]. Biosynthesis and biocatalysis, using enzymatic systems, plant extracts, or microbial pathways, facilitate the formation of metallic nanoparticles, functionalized polymers, and hybrid nanostructures without the need for hazardous reducing or stabilizing agents [4,16]. Clean energy-assisted methods such as ultrasound, microwave irradiation, and visible light-mediated synthesis accelerate nucleation and morphological control under mild reaction conditions [4,11]. In addition, sustainable microfluidic platforms provide precise size control, high reproducibility, and seamless scalability, compatible with industrial manufacturing [17]. Collectively, these approaches ensure safer, energy-efficient, and regulatory-aligned production routes for green nanomaterials [1,4,11].

Nanomaterials derived from renewable resources exhibit a broad set of physicochemical and biological properties that are essential for advanced therapeutic applications [11,18]. Structural biopolymers such as cellulose, chitosan, pectin, and alginate enable the formation of hydrated three-dimensional networks with favorable charge distribution, porosity, and functional groups that support efficient drug encapsulation, colloidal stability, and controlled release modulated by the pH, ionic strength, or enzymatic activity [8,10,19]. Bioactive plant metabolites incorporated into nanoscale delivery architectures—such as polymeric nanoparticles and nanogels, lipid nanoparticles and lipid nanocapsules, layer-by-layer nanocapsules, and colloidal nanoemulsions—can impart functional attributes including antioxidant capacity, natural photosensitization, antimicrobial activity, and immunomodulation [10,20,21], particularly when the bioactive is surface-exposed or structurally integrated rather than fully sequestered in the core. Oleaginous biomaterials significantly improve the solubility, permeability, and biodistribution of hydrophobic agents through nanoemulsion and micellar structures [12,22,23]. Microbial biopolymers such as PHA, PLA, and pullulan exhibit predictable biodegradation kinetics and high batch-to-batch reproducibility, while marine-derived polymers—including chitosan, alginates, and carrageenans—provide enzyme-mediated biodegradation and strong mucoadhesion due to their high functional group density [13,14,24]. These nanostructures often respond to external stimuli such as light, temperature, reactive oxygen species, and pathological pH gradients, enabling selective activation and improving therapeutic precision [21,25,26].

The convergence of renewable nanosystems with advanced therapeutic modalities has expanded their relevance across several high-impact biomedical domains [11,18]. In oncology, biomass-derived nanocarriers have been applied to enhance tumor targeting, modulate cellular uptake, and potentiate photothermal and photodynamic responses, including under low-intensity or natural light exposure [21,27,28]. In the context of antimicrobial resistance (AMR)—recognized by the WHO as one of the leading global health threats—sustainable nanosystems enriched with bioactive metabolites have shown efficacy against pathogenic microorganisms, complex biofilms, and multidrug-resistant strains [27,29,30,31]. These materials have also been investigated in viral and respiratory infections, contributing to the improved delivery of antiviral or anti-inflammatory agents, whereas broader sustainability and policy alignment are framed within the European Green Deal context [30,32]. In emerging fungal infections, particularly those involving resistant species of *Candida*, biomass-derived nanostructures exhibit promising antifungal performance [33]. Moreover, their structural versatility supports applications in immunotherapy, combination therapy, and theranostics, underscoring the broad translational potential of renewable nanotechnology [11,34].

The development of renewable nanomaterials is strongly aligned with the principles of the bioeconomy and circular economy, which advocate for the high-value utilization of biological resources within low-impact, resource-efficient production cycles [1,6]. The conversion of lignocellulosic residues, plant byproducts, microbial metabolites, and marine biomass into high-performance nanomaterials contributes to circular value chains that reduce petrochemical dependences and minimize ecotoxic waste generation [6,13,35]. These approaches promote decentralized, cost-effective, and sustainable production models, particularly relevant for emerging economies and resource-limited regions [1,4,6]. By integrating renewable feedstocks, low-energy processes, and biodegradable outputs, sustainable nanotechnology reinforces the convergence of health, environmental stewardship, and economic resilience [1,5,6].

Despite significant progress, the field still faces scientific and translational gaps that limit broader implementation [1,4]. A lack of standardized methodologies in green synthesis, physicochemical characterization, and biological evaluation hampers comparability and reproducibility across studies [1,4,5]. Correlations between molecular structure, nanoarchitecture, and therapeutic mechanisms remain insufficiently explored, hindering the predictive modeling of clinical performance [1,21]. Industrial scalability poses challenges as many sustainable processes remain restricted to laboratory conditions or require optimization to achieve economic viability [1,9,17]. Environmental nanotoxicology, long-term biodegradation, and the ecological impacts of nano-enabled residues require systematic investigation [1,5,26]. These gaps reinforce the need for integrative reviews aimed at consolidating current knowledge, identifying emerging trends, and outlining strategic pathways for advancing safe, efficient, and sustainable therapeutic nanotechnologies [1,4,18].

**Figure 1:**
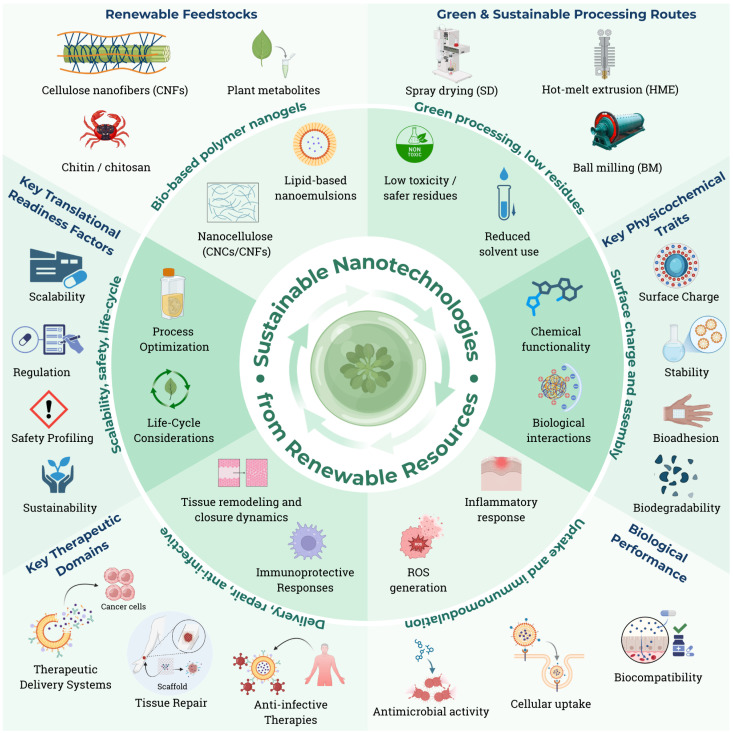
Central integrated schematic overview of sustainable nanotechnologies from renewable resources. Renewable biological feedstocks are converted through green and sustainable processing routes into bio-based polymer nanogels and related nanosystems. The resulting platforms exhibit key physicochemical traits (e.g., surface charge, stability, bioadhesion, biodegradability) that regulate biological performance, including biocompatibility, cellular uptake, antimicrobial activity, immunomodulation, and cytotoxic and inflammatory responses. Together, these properties enable applications across major therapeutic domains and inform key translational readiness factors such as scalability, regulatory compliance, safety profiling, and lifecycle sustainability.

In this review, we provide a structured evidence map of nano-enabled drug delivery and therapeutic platforms derived from renewable biological resources. We aim to (i) identify and classify nanoplatform classes and renewable feedstocks; (ii) summarize reported pharmaceutical critical quality attributes (CQAs), performance, and safety endpoints; and (iii) appraise how “renewability” and “green” claims are evidenced (feedstock origin vs. process sustainability) and how frequently translational readiness factors (scalability, quality control, regulatory alignment) are addressed. A central integrated schematic overview of these interrelated dimensions is presented in Figure 1.

## 2. Materials and Methods

This review was conducted using an integrative systematic framework designed to combine the breadth of narrative synthesis with the methodological rigor of systematic reviews. The approach followed a hybrid structure based on the PRISMA 2020. Statement, the Joanna Briggs Institute (JBI) guidelines for evidence synthesis, and the SANRA scale for assessing the quality of narrative reviews [11,18,36]. However, this work was not designed or registered as a formal systematic review or quantitative meta-analysis. Instead, elements from PRISMA 2020 and related frameworks were selectively adapted to strengthen the transparency in the search, screening, and reporting procedures, while preserving the flexibility required for a mechanistic and translational synthesis [11]. This methodological design enables the inclusion, comparison, and critical evaluation of diverse evidence types—including mechanistic studies, physicochemical characterization reports, preclinical investigations, and translational analyses—while ensuring transparency, reproducibility, and a reduced risk of bias [18].

A comprehensive literature search was performed across four primary scientific databases: PubMed/MEDLINE, the Web of Science Core Collection, Scopus, and ScienceDirect (last search: 11 February 2026). Search strings combined controlled vocabulary (MeSH, where applicable) and free-text keywords related to renewable/bio-based resources and sustainable processing, together with nanotechnology-enabled drug delivery and therapy. Database-specific queries, search fields, and retrieval counts are provided in Supplementary Table S1. No a priori publication year restriction was imposed; the final deduplicated corpus spanned 1996–2026.

In brief, the core Boolean structure was (“renewable resources” OR “biomass-derived materials” OR “biobased” OR “bioeconomy” OR “green chemistry”) AND (“nanotechnology” OR “nanomaterials” OR “nanocarriers” OR “drug delivery” OR “nanomedicine”) AND (“sustainable” OR “eco-friendly” OR “biodegradable” OR “low-toxicity”) AND (“pharmaceutical” OR “therapeutic” OR “biomedical applications”).

The reference lists of key publications were manually screened to identify additional relevant articles [18]. Records were exported to Zotero for reference management and deduplication (matching by DOI, title, and bibliographic metadata; duplicate records removed, n = NR), yielding 237 records after structured refinement [37].

Studies were considered eligible if they met the following inclusion criteria:

- Described the development, characterization, or application of nanomaterials derived from renewable resources;
- Reported pharmacotechnical, biomedical, or mechanistic data relevant to drug design, delivery, or therapy;
- Employed sustainable or green synthesis approaches, including solvent-free processes, biogenic reduction, microfluidics, or low-energy methods [4,11,16,17,21];
- Provided sufficient methodological detail for extraction and comparative analysis.

Exclusion criteria included the following:

- Studies focused exclusively on synthetic or petrochemical nanomaterials with no renewable components;
- Papers lacking primary data (e.g., opinion pieces, editorials);
- Articles with insufficient methodological description or internal inconsistencies;
- Non-pharmaceutical applications (e.g., agriculture, energy, materials science) unless they contained transferable mechanistic insights.

**Figure 2:**
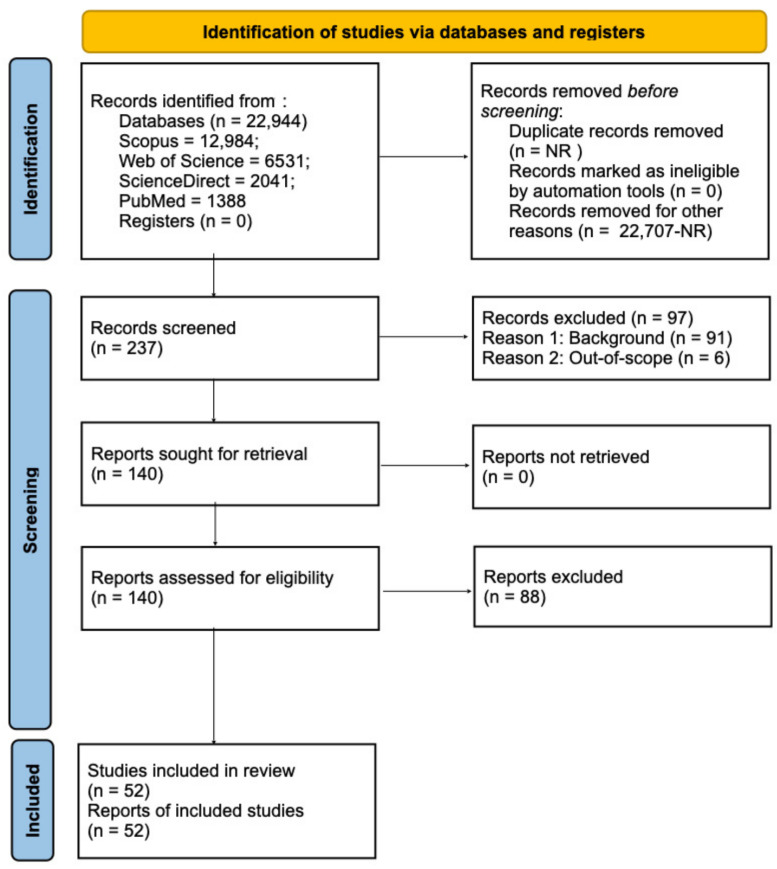
PRISMA 2020-adapted flow diagram (databases only) summarizing record identification, reference management and deduplication in Zotero, screening, full-text eligibility assessment, and final inclusion. From 22,944 records identified across Scopus, Web of Science, ScienceDirect, and PubMed, 22,707 records were removed before screening, including duplicate records (n = NR), records marked as ineligible by automation tools (n = 0), and records removed for other reasons (n = 22,707−NR). A total of 237 records were screened; 97 records were excluded at screening (background, n = 91; out-of-scope, n = 6). Full-text reports were sought for retrieval and assessed for eligibility (n = 140), and 88 reports were excluded with documented reasons (Supplementary Table S2). Studies included in the review were n = 52. Adapted from [11].

Records were screened in two stages. Titles and abstracts were assessed against the eligibility criteria, followed by a full-text evaluation of potentially relevant studies; reasons for exclusion at the full-text stage were recorded for PRISMA reporting [11]. For evidence map construction, screened records were classified into three mutually exclusive groups based on title/abstract information [20,21,38]: (i) a core nano set, comprising studies reporting nano-enabled drug delivery/therapy explicitly linked to renewable or bio-based feedstocks and/or providing evidence of green processing or upcycling; (ii) a background non-nano set, comprising biomedical/drug delivery studies centered on renewable polymers or biomaterials but without clear nanoplatform evidence in the title/abstract; and (iii) an excluded set, encompassing out-of-scope records (e.g., energy/materials, adsorption, or packaging-dominant topics without a clear pharmaceutical or therapeutic interface). The distribution of nanoplatform classes, dominant renewable feedstocks, therapeutic domains, and evidence types across the screened core set (n = 140) is summarized in Table 1; the final included studies (n = 52) were those confirmed with full-text eligibility (Figure 2). Table 1 reports screening-level evidence map descriptors for the core set retained after title/abstract screening (n = 140), whereas the final included studies (n = 52) were those confirmed after full-text eligibility assessment (Figure 2).

Data extraction was performed using a structured template that captured the source and type of renewable feedstock; the synthesis route and processing conditions; key physicochemical parameters (particle size, polydispersity index, zeta potential, and morphology); biocompatibility and biodegradation profiles; drug loading/encapsulation metrics and release mechanisms [8,18]; therapeutic indications, including oncology [11], antimicrobial resistance (AMR) [27], viral infections [30], and fungal diseases [33]; and translational, regulatory, and sustainability-related considerations.

**Figure 3:**
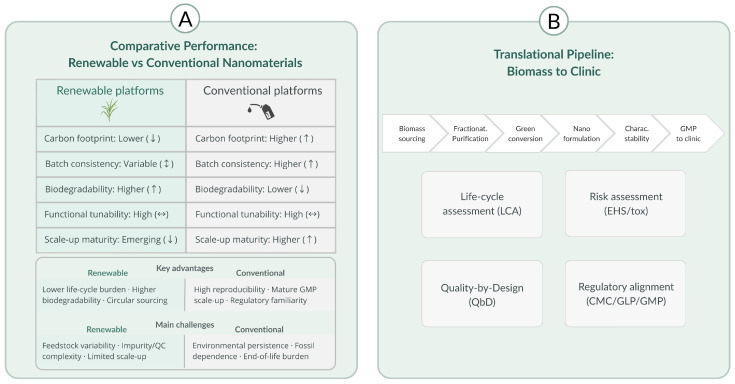
Integrated framework and mechanistic nano–bio interaction pathways for sustainable nanotechnologies. (**A**) Integrated framework connecting renewable biomass polymer building blocks (e.g., polysaccharides, chitin/chitosan, lignin motifs) to eco-efficient conversion routes (e.g., mechanochemistry, solvent-minimized processing, heterogeneous catalysis) and representative bio-based nanoplatforms (nanogels/hydrogels, engineered nanoparticles, lipid nanocarriers). (**B**) Mechanistic nano–bio interaction pathways in a target therapeutic cell context, illustrating endocytic uptake, endosomal trafficking/escape, intracellular release, ROS-related effects, mitochondrial targeting, and immunomodulatory outputs, supporting preferential action on diseased tissues with minimal toxicity to healthy cells. Arrows indicate directional relationships and process progression within the framework, including the sequential steps of the translational pipeline from biomass sourcing to clinical implementation.

To comparatively evaluate translational readiness across renewable material classes, we operationalized a domain-based appraisal aligned with CMC and scale-up expectations. For each record, we coded whether (and how explicitly) the study addressed (1) feedstock control and impurity management (provenance, fractionation/purification, compositional specifications); (2) manufacturability and scalability (process type, scale-up discussion, continuous-flow/microfluidic compatibility); (3) minimal CQA reporting and stability (size distribution/PDI, surface charge, morphology, loading/release, storage stability); (4) safety, biodegradation, and immunocompatibility (acute/subacute endpoints, degradation discussion, relevant biointerfaces); and (5) regulatory alignment signals (QbD/CMC language, sterility/endotoxin control, excipient precedent, GMP/GLP considerations). Coding categorized these aspects as not addressed, mentioned qualitatively, or supported by quantitative/validation-level data, and patterns were synthesized at the level of the nanoplatform class to support the comparative discussion in the Translational Challenges section and Figure 3. Extracted data were synthesized through an integrative thematic process comprising the structural categorization of renewable resources (lignocellulosic, metabolic plant, oleaginous, microbial/marine) [8,13,18], the classification of synthesis methodologies (green chemistry, biogenic synthesis, low-energy activation, microfluidic processing) [2,16,17], the comparative mapping of physicochemical and biological properties, and alignment with therapeutic domains of the highest global relevance, including oncology, infectious diseases, AMR, and advanced combination therapies [11,21,30].

Because the included records encompassed heterogeneous study designs—ranging from physicochemical characterization and in vitro mechanistic investigations to in vivo preclinical models and early translational reports—no single standardized risk-of-bias or quality scoring tool was uniformly applicable to all studies [39,40,41,42,43]. Instead, methodological robustness, potential sources of bias, and translational relevance were appraised qualitatively during data extraction and synthesis [18,43]. These aspects were explicitly considered in the narrative interpretation of the findings, rather than being summarized in a single quantitative quality score. Rather than aiming at the exhaustive quantitative aggregation of outcomes, this integrative structure enables a comprehensive and mechanistically grounded synthesis, providing both a panoramic overview and a detailed technical interpretation of sustainable nanotechnologies from renewable resources for advanced drug design, delivery, and therapy [18].

## 3. Renewable Resources for Sustainable Nanotechnologies

Study selection and record disposition were documented using a PRISMA 2020-adapted flow diagram (Figure 2). A total of 22,944 records were identified across Scopus, Web of Science, ScienceDirect, and PubMed (Supplementary Table S1) and imported into Zotero for reference management, deduplication, and structured thematic refinement [37]. After this workflow, 237 records were retained for title/abstract screening; 97 records were excluded at screening (background, n = 91; out-of-scope, n = 6) [21,39,41]; and 140 reports proceeded to full-text eligibility assessment. At the full-text stage, 88 reports were excluded with documented reasons (Supplementary Table S2), resulting in 52 included studies in the final evidence map. When additional full-text inspection was required to determine eligibility in ambiguous cases, the final classification was based on confirmation of nanoplatform features and explicit linkage to renewable or bio-based feedstocks and/or documented green processing evidence [11].

Renewable biological feedstocks constitute the foundational framework for sustainable nanotechnology, enabling the development of functional nanomaterials with high biocompatibility, intrinsic bioactivity, and environmentally responsible lifecycles. These feedstocks are derived from four major biomass categories—lignocellulosic, metabolic plant, oleaginous, and microbial/marine sources—each providing distinct structural motifs, chemical functionalities, and physicochemical properties essential for advanced nanomedical engineering [8,13,18]. Their integration into drug delivery platforms aligns with green chemistry principles [1,2,4] and supports circular bioeconomic models that reduce petrochemical dependences and ecotoxicological impacts [1,6,35]. The subsections below summarize the mechanistic and molecular bases that justify the use of these renewable resources in nanotechnology.

### 3.1. Lignocellulosic Biomass: Cellulose, Hemicellulose, and Lignin

Lignocellulosic biomass represents the most abundant renewable resource on Earth and comprises three principal components: cellulose, hemicellulose, and lignin [8,9]. Cellulose nanocrystals (CNCs) and nanofibrils (CNFs) derived from plant fibers exhibit exceptional mechanical strength, high surface areas, tunable charge densities, and robust colloidal stability, which make them attractive for controlled drug release, hydrogel engineering, and mucosal delivery [8,9]. Their abundant hydroxyl groups allow for surface functionalization without the need for toxic reagents, facilitating the incorporation of targeting ligands, stabilizers, or bioactive compounds [8,9]. Hemicellulose contributes amorphous, hydrophilic matrices that enhance swelling behavior, pH responsiveness, and biodegradability, while lignin provides aromatic structures with intrinsic antioxidant and UV-protective properties [8]. Together, these components form a versatile class of eco-friendly nanomaterials with demonstrated potential in drug solubilization, sustained release, and combination therapy platforms [8,9].

### 3.2. Metabolic Plant Biomass: Phytochemicals, Polyphenols, Curcuminoids, Terpenoids, and Alkaloids

Metabolic plant biomass encompasses a wide spectrum of secondary metabolites—polyphenols, flavonoids, curcuminoids, quinones, terpenoids, and alkaloids—that function as renewable molecular building blocks for nanostructure assembly [6,11]. These phytochemicals possess intrinsic pharmacological properties, including antioxidant, antimicrobial, anti-inflammatory, and natural photosensitizing properties [11,21,30]. Their multifunctionality enables the design of hybrid nanosystems that combine structural support with therapeutic bioactivity, reducing the need for synthetic excipients [4,11]. Polyphenols such as tannic acid and catechins can mediate nanoparticle self-assembly through hydrogen bonding and metal coordination [11], while curcuminoids and quinones introduce conjugated π–systems that facilitate photodynamic and redox-based mechanisms relevant to oncology and antimicrobial therapy [10,21]. Terpenoids and alkaloids contribute molecular scaffolds with membrane-permeabilizing, immunomodulatory, and antiparasitic properties, broadening the therapeutic applicability of plant-derived nanosystems [29,30]. Collectively, metabolic plant biomass provides a chemically rich platform for eco-friendly nanomaterials with built-in pharmacodynamic functionality [11,18].

### 3.3. Oleaginous Biomass: Essential Oils, Plant Lipids, and Amphiphilic Constituents

Oleaginous biomass—including essential oils, triglycerides, sterols, and amphiphilic plant lipids—serves as a renewable source for nanoemulsions, micelles, nanocapsules, and other self-assembled lipidic structures, widely used in pharmaceutical delivery [12,18,22]. Essential oils provide volatile terpenoids with antimicrobial, anti-inflammatory, and permeation-enhancing activity and are frequently formulated into nanoemulsions, lipid nanocapsules, solid–lipid nanoparticles/nanostructured lipid carriers, or polymeric nanocapsules to create dual-function systems in which the carrier architecture enhances delivery while the terpenoid fraction contributes direct bioactivity [29,30]. Plant-derived lipids can form stable colloidal nanocarriers—including nanoemulsions (oil droplets stabilized by surfactants/biopolymers), vesicular systems such as liposomes/phytosomes, solid–lipid nanoparticles, nanostructured lipid carriers, and lipid nanocapsules—exhibiting high biocompatibility, favorable membrane interaction profiles, and the improved solubilization of hydrophobic APIs [12,22]. Their amphiphilic organization allows precise control over the droplet size, interfacial tension, and release kinetics [12]. Furthermore, many oleaginous constituents display endogenous bioactivity that enhances therapeutic synergy, particularly in combination with photosensitizers, antioxidants, or antimicrobial agents [21,30]. As such, oleaginous biomass forms a critical component of sustainable nanotechnology for improving drug bioavailability and enabling multimodal therapeutic responses [12,18].

### 3.4. Microbial and Marine Biomass: PHA, PLA, Pullulan, Chitosan, Alginate, and Carrageenan

Microbial and marine biomass constitute renewable sources of structurally diverse biopolymers, including polyhydroxyalkanoates (PHA), polylactic acid (PLA), pullulan, chitosan, alginate, and carrageenan [13,24]. These polymers are significantly valued for their inherent biodegradability, high purity, batch-to-batch reproducibility, and adaptability to green synthesis processes [13,24]. PHA and PLA are biocompatible polyesters widely used as matrices for polymeric nanoparticles and nanocapsules, including long-acting nanoscale depots [24]. Separately, biomass-derived macroscale matrices such as pullulan-based films can serve as oxygen barrier carriers for sensitive bioactives and as mucosal delivery patches; when such films incorporate embedded nanoparticles/nanogels, they constitute nano-enabled composite delivery platforms rather than standalone nanomaterials [13]. Chitosan and alginate offer cationic and anionic functional groups, respectively, enabling ionic gelation, pH-responsive release, mucoadhesion, and enhanced epithelial permeability [19,24]. Marine polysaccharides such as carrageenan exhibit strong hydrophilicity, biological adhesiveness, and enzyme-mediated biodegradation, enabling temporally precise release profiles [13,14,38]. Their combined physicochemical attributes support the design of advanced nanocarriers for applications spanning oncology [11,25], antimicrobial resistance [27,29], antiviral therapy [30], and fungal infections [33]. These renewable polymers play a pivotal role in enabling scalable, biocompatible, and environmentally aligned nanotechnologies for next-generation pharmaceutical systems [13,24]. As summarized in Table 2, these four renewable feedstock classes exhibit distinct structural and functional characteristics that directly influence their applicability in pharmaceutical nanotechnology.

## 4. Green and Sustainable Synthesis Routes for Nanomaterials

Green and sustainable synthesis routes constitute the technological core of renewable-resource-based nanotechnology, enabling the production of functional nanostructures through energy-efficient, solvent-minimized, and environmentally responsible processes. These methods operationalize the principles of green chemistry [1,2,4] while providing precise control over particle nucleation, molecular assembly, and surface architectures. By replacing petrochemical solvents, hazardous reducing agents, and energy-intensive processing steps with biogenic, low-energy, and microfluidic alternatives, sustainable synthesis routes expand the applicability, safety, and regulatory compatibility of nanomaterials used in advanced pharmaceutical delivery systems [1,4]. The subsections below detail the main categories of green synthesis that underpin the field.

### 4.1. Solvent-Free and Low-Solvent Processing

Solvent-free or low-solvent techniques represent a foundational pillar of sustainable nanomaterial synthesis. Processes such as hot-melt extrusion, mechanical milling, cryogenic grinding, spray drying, and supercritical fluid processing enable the formation of nanostructures under clean, scalable, and cost-efficient conditions [4,15,18]. Hot-melt extrusion allows polymers and active pharmaceutical ingredients (APIs) to be molten and dispersed at the nanoscale without the use of organic solvents, producing stable amorphous dispersions or nanocomposites suitable for oral and transmucosal drug delivery [4,18]. Mechanical milling reduces particle sizes through shear and impact forces, enabling the fabrication of nanocrystals with improved dissolution rates and bioavailability [18]. Spray drying creates dry, flowable powders with controlled porosity, while supercritical CO_2_ techniques facilitate the formation of submicrometric particles and polymeric foams under mild conditions that eliminate solvent residues [4,15]. Collectively, these routes align with the principles of waste minimization, reduced toxicity, and low environmental persistence, while offering high throughput and industrial feasibility [1,4].

### 4.2. Biogenic and Biocatalytic Synthesis

Biogenic synthesis leverages the natural reducing, stabilizing, and templating capabilities of plant extracts, microbial metabolites, and enzymatic systems to produce nanomaterials without hazardous reagents [4,11,30]. Plant-based synthesis utilizes secondary metabolites—polyphenols, flavonoids, terpenoids, and alkaloids—as reducing agents and capping ligands for metal nanoparticles and hybrid nanostructures [11,29,30]. These molecules mediate electron transfer events that guide nucleation and growth, generating particles with high biocompatibility and tailored surface chemistry [4,11]. Microbial routes employ bacterial, fungal, or algal cultures to biosynthesize nanostructures intracellularly or extracellularly, using metabolic by-products such as proteins, polysaccharides, and reductases as natural chelators or surface stabilizers [13,24]. Enzymatic synthesis offers precise catalytic control over reaction kinetics and stereochemistry, enabling the site-specific functionalization of polymers or controlled polymerization of renewable monomers [18,24]. Biogenic synthesis is valued for its low toxicity, benign reaction conditions, and intrinsic alignment with circular bioeconomic principles [1,6,35].

### 4.3. Clean Energy-Assisted Approaches

Sustainable nanomaterial fabrication increasingly employs clean energy-assisted techniques such as ultrasonic irradiation, microwave heating, photochemical activation, and visible light-mediated synthesis [11,18]. These strategies are integral to contemporary green nanomedicine because they reduce solvent use, shorten processing times, and lower overall energy demand while supporting the preparation of bioactive nanomaterials for therapeutic use [15,18,21]. Ultrasound generates cavitation microbubbles that induce localized high-pressure and high-temperature conditions, accelerating nucleation and enhancing mass transfer under low overall energy consumption. Microwave irradiation promotes volumetric heating and rapid molecular mobility, enabling uniform reaction environments that reduce synthesis times and solvent requirements. Photochemical and visible light-driven methods allow nanoparticle formation and surface modification through controlled redox events induced by irradiation, frequently employing plant-derived chromophores or natural photosensitizers as mediators [11,18]. These approaches offer precise spatial and temporal control over nucleation dynamics, minimize side reactions, and enable lower energy inputs relative to conventional thermal methods, making them promising for scalable and eco-friendly nanomaterial production [15,18,21].

### 4.4. Microfluidic and Continuous-Flow Green Manufacturing

Microfluidics and continuous-flow technologies represent a frontier in sustainable nanomanufacturing, bridging laboratory-scale synthesis with industrial scalability [9,17]. Microfluidic platforms provide laminar flow environments that allow precise control over mixing, nucleation, and particle growth at microscale dimensions, generating nanomaterials with narrow size distributions, reproducible morphologies, and tunable surface properties [9,17]. These systems significantly reduce reagent consumption, processing times, and energy inputs while enabling the real-time monitoring of reaction parameters [17]. Continuous-flow reactors extend these advantages to larger scales, facilitating the consistent production of nanostructures under controlled hydrodynamic, thermal, and chemical conditions [9]. Their compatibility with solvent-free reactions, biogenic reducing systems, and clean energy activation positions microfluidics as a transformative tool for green nanotechnology [9,17]. Microfluidic strategies are increasingly being integrated with renewable feedstocks—including lignocellulosic derivatives, plant metabolites, and microbial polymers—to produce complex, multifunctional nanocarriers with high translational potential [13,17,18].

### 4.5. Integration of Renewable Resources into Sustainable Synthesis Frameworks

The merging of renewable feedstocks with green synthesis technologies enables the creation of nanomaterials that incorporate ecological, molecular, and therapeutic advantages in a unified platform [1,4]. Importantly, the renewable feedstock origin (what the material is derived from) is conceptually distinct from sustainable processing performance (how the nanocarrier is manufactured); a renewable source does not necessarily imply low-impact processing, and “green” claims should therefore be supported by explicit process metrics. Lignocellulosic derivatives are processed into nanocrystals or nanofibers through acid-free hydrolysis, mechanical fibrillation, or aqueous enzymatic treatments [8,9,24]. Phytochemical-rich extracts function simultaneously as reducing, stabilizing, and functionalizing agents, streamlining nanoparticle synthesis and embedding intrinsic pharmacological properties [11,27,30]. Oleaginous biomaterials serve as both reaction media and structural matrices for lipid-based nanocarriers [12,22]. Microbial and marine polymers support ionic gelation, self-assembly, and templating under mild aqueous conditions [13,14,24,38]. Integrating these renewable matrices with solvent-free, biogenic, clean energy, and microfluidic methodologies amplifies sustainability while enabling fine control over the nanostructure architecture and drug-release profiles [1,4,9,17]. This convergence positions renewable resource-based nanotechnology as a scalable, low-impact, and mechanistically rich platform for advanced drug design, delivery, and therapeutic innovation [1,4,18]. The main sustainable synthesis approaches discussed in this section are comparatively summarized in Table 3. For conceptual clarity for interdisciplinary readers, Table 4 contrasts “renewable source” attributes with “sustainable processing” metrics that should be reported to substantiate green manufacturing claims.

## 5. Physicochemical and Biological Properties of Renewable Nanomaterials

Renewable nanomaterials exhibit a versatile set of physicochemical and biological properties that distinguish them from conventional, petrochemical-based nanocarriers and position them as promising platforms for sustainable drug delivery and therapeutic innovation [18,24]. These properties emerge from the intrinsic chemical functionalities of lignocellulosic structures, phytochemical metabolites, oleaginous constituents, and microbial or marine biopolymers, which collectively enable precise control over intermolecular interactions, self-assembly, biodegradation, and biological responsiveness [8,12,13,18]. Understanding these properties is essential for predicting nanostructure behavior in physiological environments, optimizing drug loading and release kinetics, and establishing mechanistic links between material composition and therapeutic outcomes [21,24]. This section outlines the principal physicochemical and biological attributes of renewable nanomaterials that support their application in advanced pharmaceutical systems [18,24]. As summarized in Figure 3, an integrated sustainability framework (panel A) links renewable feedstocks and eco-efficient conversion routes to representative bio-based nanoplatforms [1,4], while mechanistic nano–bio interaction pathways (panel B) highlight uptake, intracellular trafficking, ROS-related processes, and immunomodulation in a target therapeutic context [21,30], emphasizing preferential action on diseased tissues with minimal toxicity to healthy cells [21,24].

### 5.1. Structural Organization, Morphology, and Supramolecular Architecture

The physicochemical behavior of renewable nanomaterials is strongly governed by their supramolecular organization and morphological characteristics [18,24]. Lignocellulosic nanocrystals and nanofibrils display rod-like or fibrillar morphologies with high crystallinity and extensive hydrogen bonding networks, contributing to excellent mechanical stability, shear thinning behavior, and tunable surface charges [8,9]. Plant-derived polyphenols and curcuminoids form π–stacked assemblies stabilized by hydrophobic interactions, hydrogen bonding, and metal coordination, enabling the creation of hybrid nanostructures with photoreactive and antioxidant functionalities [11,21,27]. Oleaginous colloidal nanocarriers—including droplet-based nanoemulsions, self-assembled lipid or polymeric micelles, and discrete lipid nanoparticles/nanocapsules with core–shell organization—promote the efficient solubilization of hydrophobic APIs and can enhance colloidal stability through interfacial design (surfactant/biopolymer shells, steric stabilization, and controlled surface charge) [12,22]. Microbial and marine biopolymers such as chitosan, alginate, and carrageenan facilitate hydrogel formation, ionic crosslinking, and self-assembly into polyelectrolyte complexes with adjustable porosity and mechanical resilience [13,14,24,38]. These diverse structural motifs underpin the capacity of renewable nanomaterials to modulate drug retention, tissue interaction, and controlled release profiles [18,24].

### 5.2. Surface Chemistry, Charge Distribution, and Functional Group Availability

Surface chemistry is a critical determinant of nanoparticle stability, biointerface interactions, and biological performance [18,24]. Renewable nanomaterials present rich arrays of functional groups—hydroxyl, carboxyl, amino, sulfate, and phenolic moieties—that enable covalent modification, electrostatic interactions, and ligand conjugation without requiring toxic reagents [8,13,14,24,38]. Chitosan’s primary amines impart a positive surface charge that enhances mucoadhesion and promotes tight junction modulation, increasing epithelial permeability [19,24]. Alginate and pectin possess carboxylate groups that facilitate ionic gelation, pH-responsive behavior, and the encapsulation of cationic drugs [19,24]. Lignin and polyphenols contribute phenolic hydroxyls and aromatic domains that enable metal chelation, antioxidative radical scavenging, and light-mediated activation [8,11,21]. Such chemical diversity supports the engineering of multifunctional nanoplatforms with stimuli-responsive properties tailored to the pathological microenvironment—including an acidic pH, enzymatic activity, redox gradients, and oxidative stress [21,25].

### 5.3. Biodegradability and Environmental Fate

The biodegradability of renewable nanomaterials is one of their most significant advantages over synthetic nanocarriers [13,24]. Biopolymers such as cellulose, hemicellulose, pectin, alginate, carrageenan, and microbial polyesters (PHA, PLA) undergo enzymatic or hydrolytic degradation into non-toxic, metabolizable fragments [8,13,14,24,38]. This minimizes accumulation in tissues, enables predictable clearance kinetics, and reduces long-term toxicological concerns [24,43]. Lignin and plant polyphenols degrade through oxidative and enzymatic pathways, producing low-molecular-weight aromatic compounds that enter natural biochemical cycles [8,11]. Oleaginous matrices degrade through lipolysis and β-oxidation, yielding fatty acids and glycerol [12,22], while chitosan undergoes depolymerization by lysozyme and chitinases [19,24]. These natural degradation pathways align with the principles of a circular bioeconomy and reduced environmental persistence [1,6,35], while also facilitating compliance with regulatory frameworks concerned with chronic nanotoxicity and ecological safety [5,24].

### 5.4. Drug Encapsulation, Binding Interactions, and Release Mechanisms

The interaction of renewable nanomaterials with APIs is governed by the chemical affinity between drug molecules and material components [18,24]. Hydrogen bonding, electrostatic attraction, hydrophobic packing, π–π interactions, and metal coordination all contribute to the encapsulation and retention of therapeutic agents [11,24,27]. Lignocellulosic nanofibrils immobilize hydrophilic and amphiphilic drugs through extensive hydrogen bonding networks, enabling prolonged release profiles [8,9]. Polyphenolic matrices stabilize hydrophobic drugs and natural chromophores via π–stacking, enhancing photophysical properties relevant to photodynamic therapy [11,21]. Lipid-based nanoemulsions and micelles encapsulate poorly water-soluble compounds within hydrophobic cores, enabling improved oral and topical bioavailability [12,22]. Marine polysaccharide nanogels and nano-enabled hydrogel matrices can provide ion-dependent release triggered by the local pH or ionic strength, facilitating site-specific delivery in mucosal tissues [13,14,24,38]. The controlled tuning of these intermolecular forces enables customizable pharmacokinetic profiles optimized for specific therapeutic indications [18,24]. A comparative overview of the dominant encapsulation mechanisms, release triggers, and associated pharmaceutical advantages of renewable resource-based nanomaterials is provided in Table 5.

### 5.5. Interactions with Biological Membranes and Mucosal Barriers

Biomass-derived nanocarriers can display distinct interaction patterns with cellular membranes and mucosal barriers governed by the nanoscale interfacial chemistry (surface charge, hydration, and exposed functional groups) rather than biomass origin per se [18,24]. For example, chitosan-containing nanoparticles and nanogels can enhance mucosal residence times through electrostatic interactions between cationic amino groups and anionic mucins and may modulate paracellular transport via transient tight junction regulation, reflecting the intrinsic polymer chemistry amplified by the nanoscale surface area [19,24]. Oleaginous colloidal nanocarriers (nanoemulsions, micelles, lipid nanoparticles/nanocapsules) can interact favorably with lipid bilayers due to hydrophobic compatibility, increasing membrane permeability and enabling transdermal penetration [12,22]. Polyphenol-rich coatings on nanoparticles and nanocapsules can modulate membrane fluidity and, in antimicrobial contexts, contribute to membrane disruption and synergy in combination therapies [29,30]. Lignocellulosic nanofibers and nanogels can form viscoelastic, nano-enabled matrices that mimic extracellular environments and support localized delivery and tissue adhesion [8,9,24]. These interactions collectively improve drug absorption, tissue retention, and therapeutic specificity across gastrointestinal, pulmonary, mucosal, and dermal pathways [13,24].

### 5.6. Stimuli Responsiveness and Microenvironmental Adaptation

A key functional advantage of renewable nanomaterials is their responsiveness to environmental stimuli, including pH, temperature, ionic strength gradients, oxidative stress, and light exposure [21,24,25]. pH-responsive behavior is exhibited by polysaccharides containing ionizable groups (alginate, chitosan, pectin), enabling drug release in acidic tumor microenvironments or inflamed tissues [19,24,25]. Thermoresponsive systems—including certain lignin derivatives and lipid-based carriers—alter their structural organization upon heating, enabling controlled gelation or triggered drug diffusion [12,22,24]. Polyphenolic nanoparticles respond to oxidative stress by releasing antioxidants or activating redox-dependent therapeutic pathways, enhancing their relevance in chronic inflammatory and degenerative diseases [11,21]. Curcuminoid- and polyphenol-enabled nanocarriers—such as curcumin-loaded polymeric nanoparticles or lipid nanoparticles and polyphenol-coated or polyphenol-crosslinked nanogels/nanocapsules (e.g., tannin-mediated networks)—can exhibit light-triggered excitation and ROS generation, relevant to photodynamic and, in selected contexts, photothermal applications [10,21,25], where the optical response is primarily dictated by the incorporated chromophore. These environmentally responsive features support precision therapy with minimal off-target exposure [21,24].

### 5.7. Antioxidant, Antimicrobial, and Immunomodulatory Bioactivity

Many renewable nanomaterials possess intrinsic bioactivity that complements or potentiates their roles as drug delivery platforms [18,24]. These functionalities arise from the chemical diversity of polyphenols, curcuminoids, terpenoids, tannins, β-glucans, and marine-derived polysaccharides, enabling multimodal interactions with biological systems [11,13,21]. The major categories of bioactivity include the following.

- Antioxidant activity: Polyphenolic compounds, tannins, flavonoids, and curcuminoids exhibit a strong capacity to neutralize reactive oxygen species (ROS) and modulate redox homeostasis [11,21]. These molecules can attenuate oxidative stress in inflamed tissues, reduce lipid peroxidation, and synergize with redox-dependent therapeutic pathways [11,21].
- Antimicrobial and antibiofilm effects: Essential-oil-based nanocarriers release membrane-active terpenoids capable of disrupting microbial envelopes, inhibiting quorum sensing, and impairing virulence factors [29,30]. Chitosan and related cationic polysaccharides exert direct bactericidal and fungicidal activity via electrostatic interactions with negatively charged microbial membranes, promoting structural destabilization and biofilm penetration [24,29,30].
- Immunomodulatory properties: Biomass-derived polysaccharides such as chitosan, alginate, and β-glucans enhance immune cell activation, regulate cytokine secretion, and support macrophage-driven antimicrobial responses [13,24]. These materials can also downregulate pro-inflammatory signaling pathways, including NF-κB, contributing to the restoration of homeostatic immune balance [11,13,24].
- Wound healing and tissue regeneration: Chitosan- and alginate-based hydrogels facilitate tissue repair through moisture retention, bioadhesion, and the controlled release of bioactive molecules [24,26]. Their viscoelasticity and biodegradability create a supportive microenvironment for epithelial remodeling and extracellular matrix regeneration [24,26].

The synergy among these antioxidant, antimicrobial, and immunomodulatory mechanisms enables renewable nanomaterials to function not only as passive delivery vehicles but also as bioactive therapeutic agents [18,24]. Their multimodal interactions with biological systems contribute to enhanced efficacy across a broad spectrum of pathological contexts, including oncology, antimicrobial resistance, viral and fungal infections, parasitic diseases, and chronic inflammatory disorders [11,21,29,30,33,39]. This integrated bioactivity positions renewable nanomaterials as versatile platforms capable of addressing complex therapeutic demands while maintaining favorable safety, biodegradability, and environmental profiles [13,24]. A comprehensive comparative view of the structural, physicochemical, and functional attributes of renewable resource-based nanomaterials, including their feedstocks, green synthesis routes, and drug encapsulation mechanisms, is already provided in Table 1, Table 2 and Table 3. These integrated properties underpin the biomedical performance of renewable nanosystems as detailed in the subsequent sections.

## 6. Biomedical Applications of Renewable Nanotechnologies

Renewable resource-based nanotechnologies have expanded the therapeutic landscape by integrating eco-friendly materials with advanced drug delivery principles, enabling multimodal strategies that combine biocompatibility, controlled release, intrinsic bioactivity, and microenvironment-responsive behavior [18,24]. Their applications span oncology, antimicrobial resistance (AMR), viral and respiratory infections, fungal diseases, inflammatory disorders, and emerging photonic therapies [11,30,33,39]. These platforms not only enhance the pharmacokinetic and pharmacodynamic performance of conventional drugs but also enable combination therapies, theranostic integration, and light-driven activation pathways [11,21]. This section consolidates the primary therapeutic domains in which renewable nanotechnologies have demonstrated significant translational potential [18,24].

### 6.1. Oncology and Tumor Microenvironment Modulation

Oncology represents one of the most advanced and impactful application areas for renewable nanotechnologies [11,34]. Biomass-derived nanocarriers enhance tumor targeting by exploiting multiple mechanisms: the enhanced permeability and retention (EPR) effect, ligand-mediated targeting, microenvironment-responsive release, and the modulation of oxidative and metabolic pathways [21,25,34]. Polyphenol- and curcuminoid-based nanoparticles exhibit photodynamic and photothermal capabilities, enabling minimally invasive therapies with selective activation under visible or near-infrared light [10,11,21]. Lignocellulosic nanofibers and marine polysaccharide hydrogels support the localized delivery of chemotherapeutics, reducing systemic toxicity and improving tissue retention [8,9,13,24]. Chitosan- and alginate-derived systems facilitate pH-dependent release within acidic tumor niches, while lipid-based nanoemulsions enhance the solubilization and uptake of hydrophobic anticancer drugs [12,19,22,25]. Moreover, renewable nanomaterials can modulate the tumor microenvironment by reducing oxidative stress, altering immunosuppressive pathways, and disrupting metabolic resilience [11,21]. Their low toxicity and adaptability enable integration with immunotherapy, radiodynamic therapy, combination chemotherapy, and molecularly targeted agents [11,21,34].

### 6.2. Antimicrobial Resistance (AMR) and Complex Biofilm Infections

Addressing antimicrobial resistance is a global priority, as acknowledged by the World Health Organization [11]. Renewable nanotechnologies offer a broad arsenal of physicochemical and biological mechanisms to combat resistant microorganisms and biofilms [24,29,30]. Essential oil-based nanoemulsions deliver membrane-active terpenoids directly to bacterial surfaces, disrupting lipid bilayers, inhibiting efflux pumps, and suppressing quorum-sensing communication [29,30]. Chitosan nanoparticles exert intrinsic bactericidal activity through the polycationic disruption of negatively charged microbial envelopes [24,29]. Polyphenolic carriers generate oxidative stress, destabilize virulence factors, and enhance antibiotic susceptibility [11,30]. Marine polysaccharides such as alginate and carrageenan modulate biofilm matrix architectures, enabling the deeper penetration of antimicrobials [13,14,38]. These mechanisms allow renewable nanomaterials to synergize with conventional antibiotics, reversing resistance phenotypes and enabling combination therapies with improved efficacy and reduced toxicity [29,30].

### 6.3. Viral and Respiratory Infections

Renewable nanomaterials have emerged as versatile platforms for antiviral therapy, particularly due to their capacity to modulate mucosal environments, enhance drug solubility, and stabilize labile antiviral molecules [13,30]. Chitosan nanoparticles enhance mucosal adhesion and prolong residence times in respiratory pathways, improving the delivery of nucleic acid-based antivirals and anti-inflammatory agents [24,32]. Lipid-derived nanoemulsions encapsulate hydrophobic antivirals, supporting targeted delivery to pulmonary or nasal tissues [12,22]. Polyphenolic nanocarriers inhibit viral entry and replication through interactions with viral envelope proteins and the modulation of host redox pathways [11,30]. Marine polysaccharides, including carrageenan, exhibit direct antiviral activity by blocking viral attachment and preventing internalization [13,14]. These renewable nanoplatforms demonstrate potential for respiratory infections such as influenza, RSV, and coronaviruses, functioning as both delivery systems and active antiviral agents [13,24,30].

### 6.4. Fungal and Emerging Opportunistic Infections

The rise of difficult-to-treat fungal pathogens—including *Candida auris*, *Aspergillus* spp., and multidrug-resistant yeasts—has intensified interest in renewable nanotechnologies with antifungal capabilities [33]. Biomass-derived antifungal nanosystems can act through a combination of intrinsic polymer-mediated interactions and payload-mediated chemical stressors [24,33]. Polyphenols and terpenoids (including essential oil constituents) can disrupt fungal ergosterol-associated pathways and compromise mitochondrial function [11,30]. Chitosan’s cationic polysaccharide chemistry enhances interaction with fungal cell walls, promoting structural destabilization and increased susceptibility to co-delivered antifungal drugs [24,33]. Essential oil-loaded nanoemulsions, lipid nanocapsules, and related nanocarriers can offer synergistic fungicidal effects and improved penetration into biofilm matrices while sustaining local exposure [29,30]. Marine polysaccharide nanogels and nano-enabled hydrogel dressings can support localized therapy for mucosal or dermal fungal infections by enabling sustained release and microenvironmental stabilization [13,14,24]. These renewable nanoplatforms address critical challenges in fungal therapy, including drug resistance, biofilm recalcitrance, and poor tissue penetration [24,33].

### 6.5. Parasitic and Neglected Tropical Diseases (NTDs)

Renewable nanomaterials hold significant promise for parasitic diseases such as leishmaniasis, malaria, and Chagas disease, which disproportionately affect regions where sustainable, low-cost, and biocompatible technologies are essential [11,18]. Plant-derived metabolites—including curcuminoids, flavonoids, terpenoids, and alkaloids—exhibit intrinsic antiparasitic and immunomodulatory effects [11,21]. When incorporated into nanocarriers, these phytochemicals demonstrate enhanced solubility, photostability, and controlled release, improving tissue penetration and intracellular delivery to infected macrophages [10,11].

Chitosan and alginate nanoparticles—a key class of microbial and marine biopolymers—promote phagocytic uptake and enable targeted delivery to parasitic reservoirs within macrophages, supporting improved therapeutic indices and reduced systemic toxicity [19,24]. Lipid-based nanoparticles enhance the biodistribution of hydrophobic antiparasitic drugs and facilitate combination strategies involving photodynamic activation, a modality that is being increasingly explored for intracellular parasites and cutaneous leishmaniasis [12,21,22]. Polyphenolic nanocarriers further contribute by modulating oxidative stress and regulating host immune responses, thereby limiting inflammatory tissue damage and promoting the resolution of infections [11,21].

From a socioeconomic perspective, the use of renewable feedstocks aligns with circular bioeconomy principles, enabling low-cost, regionally sourced, and environmentally responsible therapeutic platforms suitable for deployment in low-resource settings [1,6,35]. Collectively, these advantages position renewable nanotechnologies as transformative candidates for the treatment of neglected tropical diseases and other persistent parasitic infections [18,24].

### 6.6. Inflammatory, Oxidative, and Immune-Mediated Disorders

Renewable biomass-derived components used in nanocarriers include polyphenol- and tannin-rich fractions (as coatings/crosslinkers), polysaccharides such as chitosan and β-glucans (as matrices or surface ligands), and marine-derived polysaccharides (as gelling/stabilizing networks), assembled into nanostructures such as polymeric nanoparticles, nanogels, nanocapsules, and colloidal nanoemulsions [11,13,21]. The reported antioxidant, anti-inflammatory, and immunomodulatory effects can be intrinsic when the biomass-derived chemistry remains intact and accessible at the nano-interface (e.g., chitosan-mediated bioadhesion and barrier interactions; β-glucan receptor engagement; polyphenol-rich coatings providing redox activity). Meanwhile, in other cases, these effects are predominantly payload-mediated (e.g., encapsulated curcuminoids/polyphenols), with the nanostructure primarily improving solubility, stability, cellular uptake, and biodistribution [11,21]. Lipid-based colloidal nanocarriers can enhance the bioavailability of compounds used in inflammatory bowel disease, rheumatoid arthritis, and chronic skin inflammation [12,22]. Chitosan- and alginate-based nanogels/hydrogel nanoparticles and nano-enabled wound dressings can support tissue regeneration and wound healing by providing moisture retention, bioadhesion, and the controlled release of bioactive molecules [24,26]. These multimodal properties facilitate the precise therapeutic modulation of inflammatory microenvironments, supporting the development of regenerative and immunotherapeutic treatments [13,24].

### 6.7. Photodynamic, Photothermal, and Combination Therapies

Photodynamic and photothermal therapies (PDT and PTT) represent rapidly expanding fields in which renewable nanomaterials and green nanomedicine concepts are increasingly relevant [18,21]. Natural photosensitizers (NPS)—including curcuminoids, flavonoids, anthraquinones, and terpenoid chromophores—offer inherent photoreactivity, high biocompatibility, and desirable photophysical characteristics that can be further potentiated through nanoencapsulation [10,11,18]. When integrated into nanocarriers derived from lignocellulosic matrices, marine polysaccharides, or oleaginous systems, these compounds exhibit improved stability, controlled release, enhanced tissue penetration, and modulated absorption profiles, enabling activation under visible or near-infrared (NIR) light [8,9,12,13,22].

From a mechanistic standpoint, renewable nanocarriers enhance Type I and Type II photochemical pathways by improving molecular dispersion, preventing aggregation-induced quenching, and ensuring oxygen accessibility at the photosensitizer interface [18,21]. Polyphenolic and curcuminoid-based nanostructures generate reactive oxygen species (ROS), including singlet oxygen (O21), superoxide ($O_{2}^{•-}$), and hydroxyl radicals (OH^•^), which collectively induce oxidative damage to cancer cells, bacteria, fungi, or parasitic organisms [11,21,30,33]. In combination with thermogenic organic chromophores, these renewable nanocarriers also facilitate PTT by converting absorbed light into localized heat, enabling synergistic tumor ablation and enhanced microbial eradication [21].

Oleaginous nanoemulsions significantly improve the solubilization and photostability of hydrophobic photosensitizers, allowing efficient tissue penetration for topical, mucosal, or dermal PDT applications [12,22]. Chitosan- and alginate-based nanosystems support targeted delivery to infected or neoplastic tissues due to mucoadhesion, charge-mediated localization, and enzymatic responsiveness [19,24]. Marine and microbial polysaccharides further stabilize excited-state molecules and modulate oxygen diffusion, thereby enhancing ROS quantum yields and improving overall PDT efficacy [13,24].

A comprehensive comparative analysis of renewable resource-based nanomaterials, including their feedstocks, green processing routes, physicochemical attributes, and drug encapsulation mechanisms, is summarized in Table 1, Table 2 and Table 3. These structural and functional parameters underpin the biomedical performance of renewable nanosystems and dictate their suitability for targeted applications across oncology, infectious diseases, wound management, and neglected tropical conditions. To build upon these physicochemical foundations and provide a translational perspective, Table 6 organizes current renewable nanotechnologies according to disease-oriented therapeutic domains, highlighting how the material origin, delivery strategy, and mechanistic advantages converge to produce clinically relevant outcomes.

As outlined in Table 6, renewable nanoplatforms demonstrate multimodal therapeutic potential, supported by intrinsic structural, chemical, and biointeractive attributes derived from their biomass-origin components [18,24]. Their abilities to modulate redox states, enhance membrane interactions, stabilize bioactive metabolites, or sustain localized drug release directly reflect the physicochemical profiles discussed earlier [11,21,24]. Collectively, these disease-oriented applications position renewable nanotechnologies as promising candidates for next-generation therapeutic strategies that align with clinical needs for efficacy, biocompatibility, and sustainability [1,18,24].

### 6.8. Theranostics and Multimodal Imaging

Theranostic nanoplatforms derived from renewable resources have emerged as highly versatile systems capable of integrating diagnostic and therapeutic functionalities within a single molecular architecture [18,21]. Polyphenolic matrices—particularly tannins, catechins, and curcuminoids—exhibit strong metal-chelating capacities, enabling the formation of contrast-enhanced nanoclusters for optical, magnetic, or photoacoustic imaging [11,21]. These phytochemical-rich structures stabilize metal ions, modulate redox states, and improve colloidal uniformity, supporting high-resolution imaging while maintaining biocompatibility [11,21].

Lignocellulosic nanostructures, including nanocrystals and nanofibrils, provide rigid, high-surface-area scaffolds that can be functionalized with fluorescent dyes or radiolabeled probes, enabling the real-time monitoring of biodistribution, drug release, and tissue penetration [8,9]. Their abundant hydroxyl groups facilitate the covalent or hydrogen bond-mediated loading of optical reporters without the need for synthetic coupling agents [8,9]. Marine polysaccharides such as alginate, carrageenan, and chitosan form hydrogels and nanogels capable of hosting fluorescent, photoactive, or radiotracer molecules, offering controlled-release kinetics, enhanced tissue adhesion, and predictable biodegradation pathways [13,14,24,38].

When combined with renewable photoactive compounds—including curcuminoids, quinones, and flavonoids—these nanomaterials enable integrated imaging and therapeutic activation [21]. Light-triggered pathways allow the simultaneous monitoring of nanoparticle accumulation and the activation of photodynamic or photothermal effects [21,25]. This dual functionality is particularly advantageous in oncology, antimicrobial resistance, and fungal infections, where multimodal imaging enhances therapeutic precision and reduces off-target toxicity [21,27,33]. In infectious diseases and antiviral therapy, renewable theranostic systems facilitate the visualization of mucosal distribution and therapeutic uptake, improving the targeting of respiratory and epithelial tissues [24,30].

Collectively, renewable resource-based theranostic nanomaterials provide a biocompatible platform for precision medicine, integrating imaging, drug delivery, and light-driven therapeutic activation into unified, biodegradable nanosystems with reduced environmental persistence and enhanced translational potential [18,24]. A consolidated overview of the imaging modalities, mechanistic bases, and theranostic functionalities enabled by renewable nanotechnologies is presented in Table 7.

## 7. Translational Challenges and Regulatory Considerations

**Figure 4:**
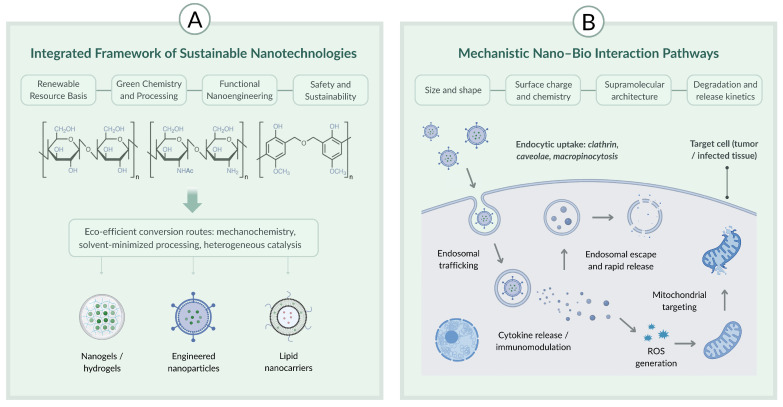
Comparative performance and translational pathway for renewable versus conventional nanomaterials in pharmaceutical development. (**A**) Comparative performance map summarizing typical trade-offs between renewable and conventional platforms across carbon footprint, batch consistency, biodegradability, functional tunability, and scale-up maturity. (**B**) Translational pipeline from biomass sourcing to clinical implementation, highlighting the main unit operations (biomass sourcing, fractionation/purification, green conversion, nanoformulation, characterization/stability, and GMP scale-up/clinical translation) and the cross-cutting enablers required throughout development, including lifecycle assessment (LCA), EHS/toxicology risk assessment, quality-by-design (QbD), and regulatory alignment (CMC/GLP/GMP).

The translation of renewable resource-based nanotechnologies into clinically applicable pharmaceutical products is constrained by scientific, technological, regulatory, and socioeconomic barriers [1,4,24]. Although biomass-derived nanomaterials offer inherent advantages in terms of biocompatibility, biodegradability, and alignment with circular bioeconomy principles, their path to industrial-scale production and regulatory approval remains complex [1,6,35]. This section summarizes the major translational challenges and outlines strategic directions to advance renewable nanotechnologies toward real-world therapeutic implementation [18,24]. As summarized in Figure 4, the key performance trade-offs and the translational pathway from biomass sourcing to clinical implementation can be rationalized as a stepwise pipeline supported by cross-cutting enablers (LCA, EHS/tox, QbD, and regulatory alignment) [1,24].

A structured overview of the principal translational barriers and regulatory challenges affecting renewable nanomaterials is presented in Table 8.

### 7.1. Scalability, Manufacturing, and Reproducibility

Renewable nanomaterials present unique challenges for large-scale manufacturing due to the chemical diversity and natural variability of biomass-derived feedstocks [5,6,35]. Achieving pharmaceutical-grade reproducibility requires addressing several critical factors.

- Batch-to-batch variability: Differences in lignin content, essential oil chemotypes, and polyphenolic composition influence nanoparticle formation, morphology, surface charge, and encapsulation efficiency, ultimately affecting therapeutic performance [8,11,22].
- Feedstock heterogeneity: Seasonal, geographic, and environmental conditions alter the chemical profiles of renewable inputs, necessitating standardized protocols for extraction, purification, and storage to minimize compositional drift [5,6].
- Process scalability: Mechanical fibrillation, biogenic synthesis, and nanoemulsification methods often require re-optimization when transitioned from bench to pilot or industrial scale to preserve particle homogeneity, stability, and functional properties [1,4].
- Controlled manufacturing environments: Advanced platforms such as microfluidic mixers and continuous-flow reactors enhance control over mixing, nucleation, and growth processes, improving size distribution and reproducibility but demanding specialized infrastructure and expertise [9,17].
- Economic and logistical constraints: Despite the nominal abundance and low intrinsic cost of biomass, upstream processing, quality control, and waste management can increase manufacturing costs, particularly in regions without established biorefinery networks [6,35].

To strengthen reproducibility and CMC transferability, a minimal standardized characterization set for renewable nanocarriers should include (i) size distribution and polydispersity (DLS and, where appropriate, nanoparticle tracking analysis); (ii) surface charge (zeta potential in relevant media); (iii) morphology and core–shell organization (TEM/cryo-TEM/AFM/SEM as applicable); (iv) chemical composition and surface functionality (FTIR/NMR and, when needed, XPS, plus crystallinity for CNC/CNF via XRD); (v) drug loading/encapsulation and free-drug fraction; (vi) release kinetics under biorelevant conditions with mechanism-aware fitting; (vii) colloidal and chemical stability (accelerated and long-term; protein corona sensitivity where relevant); and (viii) safety-critical controls including sterility/endotoxin burden, residual solvents, and key impurities/co-extractives. For photoactive systems, the minimal set should additionally report absorbance/emission profiles and ROS-related photochemical performance metrics.

Addressing these challenges requires integrated process-analytical technologies, robust quality-by-design frameworks, and coordinated efforts between academia, industry, and policymakers to develop scalable and economically viable production routes for renewable nanomaterials [1,4,24].

### 7.2. Regulatory Frameworks for Natural and Renewable Nanomaterials

The current regulatory frameworks were largely developed for synthetic nanocarriers and conventional excipients and therefore do not fully capture the specificities of renewable resource-based nanomaterials [1,4,24]. This misalignment generates uncertainty in dossier preparation and slows clinical translation [24]. Major regulatory challenges include the following.

- Absence of dedicated regulatory pathways: Broad nanotechnology guidance often does not explicitly address the natural variability, multifunctionality, or complex biodegradation kinetics characteristic of biomass-derived systems [24].
- Stringent purity and safety requirements: Even when derived from edible or traditionally used biomaterials, renewable nanocarriers must meet rigorous specifications for residual metabolites, endotoxins, bound metal ions, solvent traces, and potential immunogenic impurities [13,24].
- Complex physicochemical characterization: Regulators require detailed, multiparametric characterization of size distribution, morphology, surface chemistry, polydispersity, crystallinity, and degradation behavior, often demanding advanced analytical platforms such as NMR, FTIR, LC-MS, GC-MS, TEM, and DLS [5,24].
- Toxicological uncertainty: Biodegradation yields low-molecular-weight fragments (e.g., sugars, organic acids, phenolic derivatives) whose systemic and environmental safety must be evaluated through acute, subacute, chronic, and ecotoxicological studies [5,24].
- Reproducibility and quality control: Demonstrating batch consistency is challenging in the presence of feedstock variability, requiring the robust standardization of upstream biomass processing and downstream nanoformulation steps [5,24].
- Environmental risk assessment: Increasingly, environmental persistence, ecotoxicity, and interactions with wastewater treatment systems must be considered, particularly for platforms destined for chronic or large-scale use [5,24].

The development of harmonized, risk-based regulatory guidelines tailored to renewable nanomaterials will be essential in accelerating their clinical translation [1,4,24].

### 7.3. Toxicological Assessment and Biodegradation Pathways

Despite their generally favorable safety profiles, renewable nanomaterials require comprehensive toxicological assessment due to the chemical complexity and multifunctionality of their constituents [5,24]. Critical considerations include the following.

- Chemical complexity and compositional variability: Biomass-derived matrices contain diverse phytochemicals, polysaccharides, lipids, and trace components whose biological effects may be synergistic, antagonistic, or context-dependent [11,13,22].
- Immune and inflammatory responses: Renewable polymers and metabolites can modulate innate and adaptive immune responses, influencing cytokine secretion, macrophage activation, and complement pathways, which must be characterized to distinguish therapeutic immunomodulation from adverse immunotoxicity [13,24].
- Degradation products and metabolic fate: Biodegradation generates smaller molecules that can enter systemic circulation or be excreted via renal, biliary, or fecal routes; their accumulation, metabolism, and potential organ-specific toxicity require dedicated pharmacokinetic and toxicokinetic studies [24].
- Environmental fate and ecotoxicity: Although biodegradable, these nanomaterials and their degradation intermediates may interact with aquatic and soil microbiota, requiring assessments of ecotoxicological endpoints and trophic transfer [5,6,35].
- Nanostructure–cell interface interactions: Surface charge, hydrophobicity, rigidity, and aspect ratios influence cellular internalization, endosomal escape, and potential cytotoxicity; cationic carriers (e.g., chitosan-based) may cause membrane perturbation at high concentrations [19,24].
- Genotoxicity and oxidative stress: While many renewable nanomaterials exhibit antioxidant effects, under certain conditions, they can modulate ROS generation, necessitating genotoxicity testing and oxidative stress profiling [11,21,24].

Mechanistically, degradation intermediates can shape biological responses beyond simple clearance. For example, acidic oligomers and organic acids released from polyester-like domains may locally decrease the pH and influence endo-/lysosomal stress, while phenolic fragments from lignin-/polyphenol-rich matrices can remain redox-active, chelate metal ions, and modulate cellular antioxidant programs (e.g., Nrf2/Keap1 signaling) or inflammatory pathways (e.g., NF-κB and inflammasome-associated responses) depending on the exposure context. Likewise, sugar- and uronate-rich oligosaccharides generated from polysaccharide networks may engage pattern recognition receptors and alter macrophage polarization, highlighting that degradation profiling should be coupled to pathway-aware immunotoxicity and redox assays rather than being limited to mass loss kinetics.

Establishing standardized toxicological frameworks that integrate in vitro, in vivo, and in silico approaches will be fundamental in de-risking renewable nanomaterials and supporting regulatory approval [5,24].

### 7.4. Standardization, Characterization, and Quality Control

The lack of harmonized standards for renewable nanomaterials poses a major barrier to their clinical translation and industrial adoption. Because biomass-derived systems are inherently heterogeneous, rigorous analytical and quality control strategies are required [5,24]. Key challenges include the following.

- Feedstock standardization: Variability in botanical origin, harvest time, processing, and storage conditions impacts the chemical profiles of extracts, oils, and polymers, necessitating the specification of acceptance criteria for raw materials [5,6].
- Advanced analytical characterization: Comprehensive physicochemical profiling demands multi-technique workflows combining spectroscopic (FTIR, Raman, NMR), chromatographic (HPLC, LC-MS, GC-MS), and microscopic (TEM, SEM, AFM) methods, together with DLS for hydrodynamic size and zeta potential assessment [5,24].
- Lack of universal quality metrics: Internationally accepted standards for the purity, impurity profiles, chemical fingerprints, and performance attributes of renewable nanocarriers are not yet established, hindering inter-laboratory comparability [5,24].
- Batch consistency and scale-up: Process parameters optimized at a small scale may not translate linearly to industrial reactors, requiring continuous monitoring and adjustment to maintain consistent particle sizes, polydispersity, and drug loading [9,17,24].
- Stability and shelf-life: The susceptibility of phenolics, lipids, and other labile components to oxidation, hydrolysis, or photodegradation demands stability studies under ICH-like conditions, including evaluations of functional performance over time [11,22,24].
- Analytical method validation: For regulatory acceptance, all critical assays used in quality control must be validated for accuracy, precision, linearity, robustness, and limits of detection and quantification [24].

The development of consensus guidelines and reference materials for renewable nanomaterials would substantially strengthen their translational prospects [5,24].

### 7.5. Environmental and Socioeconomic Sustainability

Renewable resource-based nanotechnologies are closely aligned with global sustainability and circular bioeconomy agendas, but their real-world deployment must be critically evaluated in terms of environmental and socioeconomic impacts [1,6,35]. Key aspects include the following.

- Sustainable biomass sourcing: Large-scale harvesting of plant, algal, or marine biomass must be managed to avoid ecosystem degradation, biodiversity loss, and competition with food or traditional uses [6,35].
- Carbon footprint and energy demand: Green synthesis methods can reduce energy consumption and greenhouse gas emissions relative to conventional nanomanufacturing, but these gains must be quantified through lifecycle assessment [1,4].
- Environmental fate: Biodegradability generally reduces long-term persistence, yet degradation intermediates may interact with environmental microbiota and trophic networks, necessitating ecotoxicological evaluation [5,6,35].
- Circular bioeconomy integration: The valorization of agricultural, forestry, and marine residues into high-value nanomaterials strengthens circularity, reduces waste, and enhances resource efficiency [6,35].
- Access in low- and middle-income countries: The local availability of renewable feedstocks offers an opportunity for the decentralized production of nanopharmaceuticals, but this requires appropriate infrastructure, technology transfer, and regulatory harmonization [1,6].
- Economic feasibility: While raw biomass may be inexpensive, purification, standardization, and quality control introduce additional costs; economic viability depends on scalable biorefinery models and integration into existing value chains [6,35].

Embedding sustainability metrics and lifecycle thinking into early-stage research and development will be crucial to ensure that renewable nanotechnologies contribute meaningfully to equitable and resilient healthcare systems [1,6,35].

## 8. Future Perspectives and Strategic Directions

Renewable resource-based nanotechnologies are positioned to become a central pillar in next-generation pharmaceutical innovation, driven by their intrinsic biocompatibility, biodegradability, and alignment with circular bioeconomy principles [6,18,24,35]. As the field matures, future progress will depend on convergent advances in materials science, synthetic biology, computational modeling, and green biomanufacturing [1,4,9]. These developments will guide the rational design of multifunctional, sustainable, and clinically scalable nanocarriers that address global therapeutic needs [18,24].

### 8.1. Bioinspired, Biomimetic, and Modular Nanocarriers

Biological systems provide sophisticated models for structural organization, energy transfer, and molecular recognition [21]. Renewable nanomaterials offer unique opportunities for biomimetic engineering, including the following:

- Nanostructures inspired by photosynthetic antenna complexes to enhance light harvesting and energy transfer efficiency for PDT/PTT [21];
- Lignocellulosic and polyphenolic scaffolds with hierarchical architectures for controlled hydration dynamics, drug loading, and targeted release [8,9,11,24];
- Modular assemblies based on marine and microbial polysaccharides, enabling multi-therapeutic or theranostic integration [13,24];
- Hybrid renewable–synthetic constructs that combine natural biocompatibility with engineered physicochemical precision [18,24].

### 8.2. AI-Guided Design, Predictive Modeling, and In Silico Optimization

Artificial intelligence is expected to accelerate the discovery and optimization of renewable nanocarriers [5]. Key future directions include the following:

- Machine learning prediction of the photophysical properties, ROS quantum yields, aggregation tendencies, and solubility of natural chromophores [21];
- QSAR- and multi-omics-based toxicity forecasting integrated with biodegradation modeling [5,24];
- Digital twins for optimizing microfluidic and continuous-flow synthesis parameters with minimal waste generation [9,17];
- Virtual screening pipelines for selecting ideal biomass feedstocks based on chemical richness, environmental footprints, and therapeutic alignment [1,6,35].

### 8.3. Advanced Manufacturing and Scalable Green Bioprocessing

Industrial translation will rely on advanced manufacturing strategies tailored to renewable feedstocks [9,17]:

- Continuous-flow biomanufacturing to improve reproducibility, reduce solvent use, and minimize energy consumption [9];
- Microfluidic-assisted nucleation for fine control of nanoparticle formation and size distribution [9,17];
- Integrated biorefinery models capable of transforming agricultural, marine, or forestry residues into pharmaceutical-grade nanocarriers [6,35];
- Regulatory-aligned quality-by-design (QbD) frameworks guiding scale-up, validation, and quality control [24].

### 8.4. Multimodal, Light-Responsive, and Precision Therapeutic Platforms

Renewable nanomaterials are ideally suited for emerging therapeutic modalities based on natural photosensitizers, redox-active metabolites, immunomodulators, and bioactive polymers [11,13,21]:

- Daylight-activated PDT systems enabling treatment in outpatient or resource-limited settings [21];
- Combination PDT/PTT/immunotherapy platforms leveraging synergistic ROS and photothermal responses [21];
- Nanocarriers enabling simultaneous antimicrobial, antifungal, antiparasitic, and anticancer actions [11,29,30,33];
- Renewable theranostic platforms incorporating fluorescence, photoacoustic, or radiolabeled modalities for real-time monitoring [11,21].

### 8.5. Sustainability, Circular Bioeconomy, and Global Health

Renewable nanotechnologies align with sustainability targets and global health priorities, particularly in low- and middle-income countries [1,6,35]:

- Circular bioeconomy strategies converting biomass residues into high-value therapeutic platforms [6,35];
- Scalable renewable nanocarriers for neglected tropical diseases, AMR, and infectious diseases prevalent in LMICs [1,6,39];
- Early integration of lifecycle assessment and environmental metrics into development pipelines [1,5,24];
- Models for decentralized, local manufacturing, supporting affordable and equitable access [1,6].

### 8.6. Overall Outlook

The convergence of biomimetic engineering, AI-driven optimization, sustainable bioprocessing, and multimodal therapeutic design positions renewable nanotechnologies at the forefront of future pharmaceutical development [9,18,21,24]. By integrating scientific innovation with environmental responsibility and global accessibility, biomass-derived nanocarriers are poised to shape the next generation of safe, effective, and sustainable therapeutic platforms [1,18,24].

The translation of renewable nanocarriers is most strongly limited by feedstock variability and impurity control, insufficiently standardized CQA reporting and stability data, scale-dependent shifts in colloidal structure during manufacturing, incomplete pathway-aware biodegradation/toxicology characterization, and the inconsistent substantiation of “green” claims beyond renewable origins. Enabling directions include domain-based translational readiness scoring aligned with CMC/QbD expectations, the adoption of a minimal standardized characterization set, continuous-flow and microfluidic manufacturing with process-analytical technologies, the harmonized reporting of sustainability metrics (LCA-informed), and early regulatory engagement to define specification strategies for biomass-derived excipients and multifunctional nanomedicines.

## 9. Conclusions

Renewable resource-based nanotechnologies represent a rapidly advancing frontier in sustainable pharmaceutical development [18,24]. The unique physicochemical, biological, and environmental advantages of biomass-derived nanocarriers—including biocompatibility, biodegradability, chemical versatility, and alignment with circular bioeconomy principles—provide a compelling foundation for next-generation drug design, delivery, and therapeutic innovation [1,6,18,24,35]. Significant progress has been made in engineering lignocellulosic nanostructures, polyphenolic platforms, oleaginous nanoemulsions, and marine or microbial polysaccharide-based systems [8,9,11,12,13,22,24], enabling advances in oncology, antimicrobial resistance, infectious diseases, inflammatory disorders, and multimodal light-responsive therapies [11,21,30,33,39].

Despite these achievements, major translational challenges remain, particularly regarding large-scale manufacturing, regulatory harmonization, toxicological evaluation, and comprehensive environmental assessment [5,24]. Addressing these gaps will require coordinated efforts across materials science, bioprocess engineering, computational modeling, and global health policy [1,4,6].

Overall, the integration of renewable feedstocks with advanced nanotechnological strategies offers a transformative pathway toward sustainable, accessible, and high-performance therapeutic platforms [1,18,24]. As research continues to expand, renewable nanotechnologies are poised to exert a substantial impact on global healthcare by bridging scientific excellence with environmental stewardship and socioeconomic responsibility [1,6,35]. 

## Figures and Tables

**Table 1:** Evidence-map summary of the screened core set (n = 140): nanoplatform classes, dominant renewable feedstocks, therapeutic domains, evidence types, and representative sources. The final included studies (n = 52) correspond to records confirmed with full-text eligibility (Figure 2).

Nanoplatform Class	n (%)	Dominant Renewable Feedstocks (Top 3)	Dominant Therapeutic Domains (Top 3)	Evidence Type	Representative Studies
Nanoparticles/nanocapsules	103 (73.6%)	Chitosan (n = 70); Proteins—albumin/zein/casein (n = 18); Gelatin/collagen (n = 8)	Unspecified (n = 29); Oncology (n = 28); Infection/antimicrobial (n = 16)	Original research (n = 94); Review (n = 8); Book (n = 1)	Yazdan (2024) [2]; Abdelhamid and Mathew (2022) [13]; Li et al. (2022) [14]
Nanofibers (electrospun)	10 (7.1%)	Chitosan (n = 3); Proteins—albumin/zein/casein (n = 3); Gelatin/collagen (n = 2)	Infection/antimicrobial (n = 2); Oncology (n = 2); Unspecified (n = 2)	Original research (n = 10)	Bertoni et al. (2022) [15]; Huang et al. (2024) [9]; Rabiee et al. (2023) [18]
Nanogels	8 (5.7%)	Chitosan (n = 3); Gelatin/collagen (n = 3); Dextran (n = 1)	Infection/antimicrobial (n = 3); Oncology (n = 3); Unspecified (n = 2)	Original research (n = 6); Review (n = 2)	Tenchov et al. (2022) [5]; Bertoni et al. (2022) [15]; Banerjee et al. (2023) [24]
Polymeric micelles	5 (3.6%)	Chitosan (n = 3); Gelatin/collagen (n = 1); Proteins—albumin/zein/casein (n = 1)	Oncology (n = 3); Unspecified (n = 1); Infection/antimicrobial (n = 1)	Original research (n = 5)	Yazdan (2024) [2]; Li et al. (2022) [14]; Huang et al. (2024) [9]
Other nanosystems	4 (2.9%)	Chitosan (n = 3); Alginate (n = 1)	Unspecified (n = 2); Infection/antimicrobial (n = 1); Oncology (n = 1)	Original research (n = 4)	Li et al. (2022) [14]; Zhou et al. (2024) [26]; Rabiee et al. (2023) [18]
Liposomes/lipid nanoparticles (LNPs)	3 (2.1%)	Chitosan (n = 2); Proteins—albumin/zein/casein (n = 1)	Oncology (n = 2); Vaccines/immunotherapy (n = 1)	Original research (n = 3)	Huang et al. (2024) [9]; Bertoni et al. (2022) [15]; Banerjee et al. (2023) [24]
Nanoemulsions	2 (1.4%)	Chitosan (n = 1); Gelatin/collagen (n = 1)	Skin/wound healing (n = 1); Infection/antimicrobial (n = 1)	Original research (n = 2)	Bertoni et al. (2022) [15]; Banerjee et al. (2023) [24]; Ho et al. (2025) [27]
Metal/metal oxide nanoparticles	2 (1.4%)	Chitosan (n = 2)	Infection/antimicrobial (n = 1); Oncology (n = 1)	Original research (n = 2)	Fahmy et al. (2020) [31]; Ho et al. (2025) [27]; Bertoni et al. (2022) [15]
Nanogels/hydrogel nanoparticles (nano-hydrogels),nano-enabled hydrogel composites	1 (0.7%)	Chitosan (n = 1)	Skin/wound healing (n = 1)	Original research (n = 1)	Zhou et al. (2024) [26]; Li et al. (2022) [14]; Bertoni et al. (2022) [15]
Carbon dots/quantum dots	1 (0.7%)	Chitosan (n = 1)	Oncology (n = 1)	Original research (n = 1)	Huang et al. (2024) [9]; Bertoni et al. (2022) [15]; Rabiee et al. (2023) [18]
Unspecified	1 (0.7%)	Pectin (n = 1)	Unspecified (n = 1)	Review (n = 1)	Bertoni et al. (2022) [15]; Banerjee et al. (2023) [24]; Tenchov et al. (2022) [5]

Note: Macroscale hydrogels, films, and implants are discussed only when they are nano-enabled (i.e., incorporate nanoscale carrier domains such as nanoparticles/nanogels) or when nanoscale structuring is the primary determinant of loading and release; otherwise, they are outside the scope of nanomaterials.

**Table 2:** Renewable feedstocks used in pharmaceutical nanotechnology, highlighting their structural components, key functional properties, and principal therapeutic applications.

Feedstock Type	Structural Components	Key Functional Properties	Therapeutic Applications
Lignocellulosic biomass	Cellulose; hemicellulose; lignin	High mechanical stability; tunable crystallinity; controlled porosity; stability for drug encapsulation; modulation of hydration and release profiles	Oncology (PDT/PTT); antimicrobial therapies; wound healing; antioxidant delivery; controlled-release systems
Metabolic plant biomass	Flavonoids; terpenoids; alkaloids; phenolic acids; curcuminoids	Intrinsic antioxidant, anti-inflammatory, photoreactive, and antimicrobial activity; ROS modulation; natural photosensitization	PDT/PTT; AMR; viral, fungal, and parasitic diseases; anti-inflammatory applications; redox-modulating therapies
Oleaginous biomass	Triglycerides; fatty acids; phospholipids; essential oils	High solubilization capacity for hydrophobic drugs; enhanced permeation and penetration; improved photostability; formation of nanoemulsions	Topical and mucosal PDT; antimicrobial formulations; dermal delivery; pain and inflammation management; lipophilic drug delivery
Microbial and marine biomass	Chitosan; alginate; chitin; β-glucans; exopolysaccharides	Mucoadhesion; biodegradability; charge-mediated targeting; immunomodulation; pH- and enzyme-responsive behavior	Oncology; AMR; NTDs (e.g., leishmaniasis); vaccine adjuvants; wound healing; immunomodulatory therapies

**Table 3:** Green and sustainable synthesis approaches for renewable resource-based nanomaterials, their alignment with green chemistry principles, major advantages, and key limitations.

Synthesis Route	Green Chemistry Alignment	Major Advantages	Principal Limitations
Biogenic synthesis (plant, microbial, enzymatic)	Use of natural reducing/stabilizing agents; low-energy transformations; minimal hazardous reagents; waste valorization	Eco-friendly; high biocompatibility; avoids toxic precursors; enables ROS-modulating and bioactive nanostructures; scalable from low-cost biomass	Batch-to-batch variability; limited control over nucleation and particle size; dependence on biomass composition; regulatory standardization challenges
Mechanical fibrillation/nanocellulose isolation	Solvent-free or aqueous processing; minimal chemical waste	High reproducibility; tunable crystallinity and morphology; good mechanical integrity; compatible with hydrogels and drug carriers	Energy-intensive pretreatment; limited solubility; surface modification often required for pharmaceutical use
Microfluidic-assisted synthesis	Reduced reagent consumption; precise mixing; continuous-flow and low-waste operation	Excellent size control; narrow polydispersity; rapid optimization; scalable; compatible with QbD/GMP manufacturing	High initial equipment cost; risk of channel fouling; engineering requirements for scale-up
Oleaginous nanoemulsification	Low-toxicity lipids; minimal organic solvents; mild homogenization	High solubilization of hydrophobic actives; enhanced permeation; improved photostability; good dermal/mucosal compatibility	Surfactant selection critical; potential oxidative instability; temperature/light sensitivity
Green solvent extraction and polymerization	Use of ethanol, glycerol, ethyl lactate, or deep eutectic solvents; reduced hazardous waste	Safe solvent profiles; high extraction yields; suitable for natural actives; sustainable solvent lifecycle	Viscosity and polarity differences can affect reaction kinetics; regulatory approval of novel green solvents may be slow

**Table 4:** Conceptual distinction between renewable feedstock origin and sustainable processing metrics for renewable nanocarrier manufacturing.

Renewable Source Attributes (Feedstock Origin)	Sustainable Processing Metrics (Manufacturing Performance)
Biomass provenance and renewability claims (residues/by-products vs. dedicated crops; regional supply chain; seasonality).	Solvent selection and hazards (water/ethanol vs. chlorinated/regulated solvents), energy intensity (temperature/pressure), and process safety.
Intrinsic compositional variability and co-extractives (metals, endotoxins, proteins, oxidizable fractions) that influence formulation behavior.	Purification burden and waste generation (number of unit operations, yield losses, E-factor/process mass intensity, wastewater load).
Circularity potential (valorization of agricultural/marine/forestry residues; biodegradability assumptions tied to composition).	Lifecycle metrics and emissions (carbon footprint, water footprint, renewable energy share), ideally quantified via LCA-informed reporting.
Biofunctionality of the renewable fraction (surface-exposed polysaccharide chemistry; redox-active phenolics) relevant to performance.	GMP/CMC compatibility: reproducibility, sterility/endotoxin control, residual solvent/impurity specifications, and suitability for continuous manufacturing/QbD.

**Table 5:** Drug encapsulation mechanisms, release triggers, and pharmaceutical advantages of renewable resource-based nanomaterials.

Nanomaterial Class	Encapsulation/Binding Mechanisms	Release Triggers and Environmental Responses	Advantages for Drug Delivery
Lignocellulosic nanomaterials (CNC, CNF)	Hydrogen bonding; electrostatic interactions; surface adsorption; entrapment in fibrillar networks	Diffusion-controlled release; pH-dependent swelling; ionic strength modulation	Prolonged release; high mechanical stability; compatibility with hydrophilic and amphiphilic APIs
Polyphenolic and plant metabolite nanostructures	π–π stacking; hydrophobic packing; hydrogen bonding; metal coordination	Light-induced activation; ROS-sensitive degradation; enzymatic cleavage by oxidases or esterases	Stabilization of hydrophobic chromophores; redox activity; suitability for photodynamic applications
Lipid-based nanoemulsions and nanoliposomes	Hydrophobic core entrapment; membrane incorporation; interfacial adsorption; micellar solubilization	Temperature-dependent fluidity; enzymatic lipolysis; pH-mediated membrane destabilization	High encapsulation efficiency; improved permeation; solubilization of poorly soluble drugs
Chitosan and alginate nanostructures	Electrostatic attraction; ionic gelation; polyelectrolyte complexation; reversible crosslinking	pH-responsive swelling; enzyme-triggered depolymerization; ion exchange-mediated release	Mucoadhesion; enhanced epithelial transport; applications in oral, nasal, rectal, and vaginal delivery
Marine or microbial polysaccharide nanogels/nano-enabled hydrogels	Network entrapment; hydrogen bonding; ionic crosslinking; water-mediated retention	Ionic strength variations; pH gradients; diffusion-controlled release; local enzymatic activity	Biocompatibility; tunable viscoelasticity; ideal for wound healing and localized therapies
Hybrid renewable nanocomposites	Combined π–π stacking, electrostatic attraction, hydrophobic packing, and interfacial entrapment	Multi-stimuli responsiveness (pH, ROS, heat, enzymes, light); structurally controlled release	Precise release modulation; synergistic properties; suitability for theranostic and multimodal platforms

**Table 6:** Disease-oriented applications of renewable nanotechnologies.

Renewable Nanoplatform	Type of Biomass/Feedstock	Therapeutic Strategy and Target Diseases	Mechanistic Advantages and Clinical Benefits
Lignin nanoparticles	Technical lignin from kraft or organosolv pulping streams	PTT/PDT and chemo-photothermal therapy; solid tumors (breast, colon, melanoma)	Photothermal conversion; ROS generation; carrier-free loading; redox modulation; improved tumor selectivity
Chitosan nanoparticles	Chitosan from crustacean shells or fungal biomass	Antimicrobial and immunomodulatory therapy; MDR bacterial infections; viral respiratory diseases	Mucoadhesion; membrane interaction; immune activation; enhanced epithelial uptake; reduced antibiotic demand
Alginate nanogels/hydrogels	Alginate from brown seaweeds	Local antimicrobial and wound healing therapy; diabetic ulcers; chronic infected wounds	Controlled local release; moist microenvironment; improved biofilm control; accelerated wound repair
Essential oil nanoemulsions	Essential oils from Lamiaceae, Verbenaceae, Asteraceae	Antimicrobial and larvicidal therapy; skin and mucosal infections; vector-borne diseases; NTDs	Improved terpenoid stability; membrane disruption; daylight activation; low-dose, eco-friendly action
Bacterial cellulose scaffolds	Microbial cellulose (e.g., *Komagataeibacter* spp.)	Topical antifungal therapy; onychomycosis; mucocutaneous candidiasis	Bioadhesive nanofibrils; tunable porosity; sustained antifungal delivery; improved local retention
Pectin nanogels	Pectins from citrus peels or apple pomace	Antiprotozoal therapy; neglected diseases such as cutaneous leishmaniasis	pH/enzyme responsiveness; tissue interaction; high local accumulation; reduced systemic toxicity
CNC-reinforced polymeric films	Cellulose nanocrystals from lignocellulosic pulps	Antimicrobial dressings; AMR-related skin and soft-tissue infections	Mechanical reinforcement; antimicrobial barrier; gradual drug release; improved infection control

**Table 7:** Theranostic and imaging capabilities of renewable nanotechnologies.

Renewable Nanoplatform	Imaging Modality Enabled	Mechanistic Imaging Basis	Theranostic or Clinical Value
Polyphenolic nanoclusters	Fluorescence; NIR imaging; radiotracer chelation	Metal chelation and π–π interactions stabilizing optical reporters; redox-responsive emission	Real-time biodistribution tracking; image-guided therapy; enhanced diagnostic resolution
Lignocellulosic nanostructures (CNC, CNF)	Fluorescence; photoacoustic imaging	Rigid, high-surface-area scaffolds, improving dye dispersion and minimizing aggregation-induced quenching	Monitoring of tissue penetration and drug release; integration with PDT/PTT activation
Chitosan and marine polysaccharide hydrogels	Fluorescence; radiotracer incorporation	Hydrogel matrices stabilizing fluorescent or radiolabeled probes; controlled reporter release	Visualization of delivery pathways; imaging-guided localization; reduced off-target toxicity
Essential oil nanoemulsions	Optical contrast; scattering-based imaging	Terpenoid-rich cores, enhancing scattering and refractive index contrast; improved chromophore stability	Guided topical or mucosal therapy; improved delineation of infected or inflamed regions
Curcuminoid-based nanocarriers	Fluorescence; ROS-linked signal modulation	Intrinsic chromophoric properties of curcuminoids; ROS-sensitive emission and excited-state stabilization	Simultaneous imaging and PDT/PTT activation; enhanced discrimination of diseased tissues
Metal-chelating phytochemical nanocomplexes	PET/SPECT; optical imaging	Polyphenolic ligands, stabilizing radiometals; redox-mediated signal enhancement	Hybrid diagnostic–therapeutic platforms, enabling precise lesion mapping and controlled activation

**Table 8:** Translational barriers and regulatory challenges associated with renewable resource-based nanomaterials.

Challenge Category	Technical Issue	Example in Renewable Nanomaterials	Regulatory or Translational Implications
Biomass variability	Seasonal, geographic, and environmental variation in chemical composition	Lignin with fluctuating monolignol ratios; polyphenols with variable antioxidant profiles	Difficulties in ensuring batch consistency; increased analytical burden
Feedstock heterogeneity	Inconsistent polysaccharide, lignin, oil, or metabolite composition	Pectin with variable degrees of esterification; chitosan with fluctuating degrees of acetylation	Reduced reproducibility; dossier uncertainty during regulatory submission
Material characterization	Need for multiparametric analysis of size, morphology, crystallinity, surface chemistry, and charge	CNC/CNF requiring TEM, AFM, DLS, FTIR, and XRD for full characterization	High regulatory demand for advanced analytics; requirement for validated methods
Toxicological evaluation	Complex biodegradation pathways producing multiple intermediates	Polyphenolic carriers generating redox-active metabolites; alginate releasing marine oligosaccharides	Need for full toxicokinetic profiling; long-term and ecotoxicity assessment
Manufacturing scalability	Scale-dependent changes in particle size, stability, and drug loading	CNC/CNF from microfluidics to pilot scale; nanoemulsions requiring high-pressure homogenization	Quality-by-design (QbD) required; risk of non-linear scale-up effects
Regulatory compliance	Absence of dedicated regulatory pathways for natural or renewable nanomaterials	Essential oil nanoemulsions; phytochemical nanocarriers	Ambiguity in product classification; need for extensive impurity and safety data
Environmental risk assessment	Uncertain environmental fate and degradation behavior of nanomaterials	Marine polysaccharide hydrogels; biodegradable cellulose derivatives	Mandatory evaluation of environmental persistence and ecotoxicity

## Data Availability

No new data were created or analyzed in this study.

## Supplementary Materials

The following supporting information can be downloaded at: https://www.mdpi.com/article/10.3390/pharmaceutics18040407/s1, Table S1: Database-specific search strategy and retrieval log (PRISMA-adapted). Last search date: 11 February 2026. Table S2: Operational reasons for non-inclusion in the core evidence-map set (n = 97) at the title/abstract screening stage (PRISMA-adapted).

## References

[1] Suksaeree J., Maneewattanapinyo P., Monton C. (2026). AI-enabled, QbD-aligned Predictive, and Sustainable Design of Natural Polymer-based Drug Delivery Systems. AAPS PharmSciTech.

[2] Yazdan M., Naghib S.M., Mozafari M.R. (2024). Polymeric Micelle-Based Nanogels as Emerging Drug Delivery Systems in Breast Cancer Treatment: Promises and Challenges. Curr. Drug Targets.

[3] Zhuang C., Shi C., Tao F., Cui Y. (2017). Honeycomb Structural Composite Polymer Network of Gelatin and Functional Cellulose Ester for Controlled Release of Omeprazole. Int. J. Biol. Macromol..

[4] Shah S.A., Sohail M., Karperien M., Johnbosco C., Mahmood A., Kousar M. (2023). Chitosan and Carboxymethyl Cellulose-Based 3D Multifunctional Bioactive Hydrogels Loaded with Nano-Curcumin for Synergistic Diabetic Wound Repair. Int. J. Biol. Macromol..

[5] Tenchov R., Sasso J.M., Wang X., Liaw W.S., Chen C.A., Zhou Q.A. (2022). Exosomes—Nature’s Lipid Nanoparticles, a Rising Star in Drug Delivery and Diagnostics. ACS Nano.

[6] Menshutina N., Fedotova O., Abramov A., Golubev E., Sulkhanov Y., Tsygankov P. (2024). Processes of Obtaining Nanostructured Materials with a Hierarchical Porous Structure on the Example of Alginate Aerogels. Gels.

[7] Liu R., Dai L., Xu C., Wang K., Zheng C., Si C. (2020). Lignin-Based Micro- and Nanomaterials and Their Composites in Biomedical Applications. ChemSusChem.

[8] Jafernik K., Ładniak A., Blicharska E., Czarnek K., Ekiert H., Wiącek A.E., Szopa A. (2023). Chitosan-Based Nanoparticles as Effective Drug Delivery Systems—A Review. Molecules.

[9] Huang L., Huang X.H., Yang X., Hu J.Q., Zhu Y.Z., Yan P.Y., Xie Y. (2024). Novel Nano-Drug Delivery System for Natural Products and Their Application. Pharmacol. Res..

[10] Valinezhad N., Talebi A.F., Alamdari S. (2023). Biosynthesize, Physicochemical Characterization and Biological Investigations of Chitosan-Ferula gummosa Essential Oil (CS-FEO) Nanocomposite. Int. J. Biol. Macromol..

[11] World Health Organization (2023). Antimicrobial Resistance (Fact Sheet). https://www.who.int/news-room/fact-sheets/detail/antimicrobial-resistance.

[12] Page M.J., McKenzie J.E., Bossuyt P.M., Boutron I., Hoffmann T.C., Mulrow C.D., Shamseer L., Tetzlaff J.M., Akl E.A., Brennan S.E. (2021). The PRISMA 2020 Statement: An Updated Guideline for Reporting Systematic Reviews. BMJ.

[13] Abdelhamid H.N., Mathew A.P. (2022). Cellulose-Based Nanomaterials Advance Biomedicine: A Review. Int. J. Mol. Sci..

[14] Li S., Zhang H., Chen K., Jin M., Vu S.H., Jung S., He N., Zheng Z., Lee M.S. (2022). Application of Chitosan/Alginate Nanoparticle in Oral Drug Delivery Systems: Prospects and Challenges. Drug Deliv..

[15] Bertoni S., Hasa D., Albertini B., Perissutti B., Grassi M., Voinovich D., Passerini N. (2022). Better and Greener: Sustainable Pharmaceutical Manufacturing Technologies for Highly Bioavailable Solid Dosage Forms. Drug Deliv. Transl. Res..

[16] Yadegari A., Akbarzadeh M., Kargaran F., Mirzaee R., Salahshoori I., Nobre M.A.L., Khonakdar H.A. (2024). Recent Advancements in Bio-Based Dielectric and Piezoelectric Polymers and Their Biomedical Applications. J. Mater. Chem. B.

[17] Baethge C., Goldbeck-Wood S., Mertens S. (2019). SANRA—A Scale for the Quality Assessment of Narrative Review Articles. Res. Integr. Peer Rev..

[18] Rabiee N., Ahmadi S., Iravani S., Varma R.S. (2023). Natural Resources for Sustainable Synthesis of Nanomaterials with Anticancer Applications: A Move toward Green Nanomedicine. Environ. Res..

[19] Ma X., Wang Q., Yang Q., Yang X., Liu X., Fu B., Cheng S., Du M. (2024). Sheep Skin Collagen Peptide Exerts an Anti-Fatigue Effect by Improving Energy Metabolism via AMPK/PGC-1α Axis and Reducing Oxidative Damage via NRF2/HO-1 Axis. Food Biosci..

[20] Patra J.K., Das G., Fraceto L.F., Campos E.V.R., Rodriguez-Torres M.D.P., Acosta-Torres L.S., Diaz-Torres L.A., Grillo R., Swamy M.K., Sharma S. (2018). Nano Based Drug Delivery Systems: Recent Developments and Future Prospects. J. Nanobiotechnol..

[21] Mostafavi E., Medina-Cruz D., Vernet-Crua A., Chen J., Cholula-Díaz J.L., Guisbiers G., Webster T.J. (2021). Green Nanomedicine: The Path to the Next Generation of Nanomaterials for Diagnosing Brain Tumors and Therapeutics?. Expert Opin. Drug Deliv..

[22] Haddadzadegan S., Dorkoosh F., Bernkop-Schnürch A. (2022). Oral Delivery of Therapeutic Peptides and Proteins: Technology Landscape of Lipid-Based Nanocarriers. Adv. Drug Deliv. Rev..

[23] Zhang J., Jiang W., Hu L., Du Q., Filipczak N., Yalamarty S.S.K., Li X. (2025). Construction and Characterization of PDA@MnO_2_-Cored Multifunctional Targeting Nanoparticles Loaded with Survivin siRNA for Breast Tumor Therapy. Pharmaceutics.

[24] Banerjee R., Kumar K.J., Kennedy J.F. (2023). Structure and Drug Delivery Relationship of Acidic Polysaccharides: A Review. Int. J. Biol. Macromol..

[25] Inoue Y., Yoshida M., Ezawa T., Tanikawa T., Arce F., See G.L., Tomita J., Suzuki M., Oguchi T. (2021). Inclusion Complexes of Daidzein with Cyclodextrin-Based Metal-Organic Framework-1 Enhance Its Solubility and Antioxidant Capacity. AAPS PharmSciTech.

[26] Zhou X., Yu X., You T., Zhao B., Dong L., Huang C., Zhou X., Xing M., Qian W., Luo G. (2024). 3D Printing-Based Hydrogel Dressings for Wound Healing. Adv. Sci..

[27] Ho C.S., Wong C.T.H., Aung T.T., Lakshminarayanan R., Mehta J.S., Rauz S., McNally A., Kintses B., Peacock S.J., de la Fuente-Nunez C. (2025). Antimicrobial Resistance: A Concise Update. Lancet Microbe.

[28] Shah D.D., Chorawala M.R., Mansuri M.K.A., Parekh P.S., Singh S., Prajapati B.G. (2024). Biogenic Metallic Nanoparticles: From Green Synthesis to Clinical Translation. Naunyn Schmiedebergs Arch. Pharmacol..

[29] Joshi R., Chandel S., Roy I., Sharma S., Sharma S., Prajapati B.G., Kapoor D.U., Alsaidan O.A. (2026). A Review on Chitosan-Based Delivery Systems for Vitamin B_12_: Advances, Challenges, and Future Prospects. Int. J. Biol. Macromol..

[30] Breijaert T.C., Daniel G., Hedlund D., Svedlindh P., Kessler V.G., Granberg H., Håkansson K., Seisenbaeva G.A. (2022). Self-Assembly of Ferria-Nanocellulose Composite Fibres. Carbohydr. Polym..

[31] Fahmy S.A., Preis E., Bakowsky U., Azzazy H.M.E. (2020). Platinum Nanoparticles: Green Synthesis and Biomedical Applications. Molecules.

[32] Borghesi S., Vergalli S. (2022). The European Green Deal, Energy Transition and Decarbonization. Environ. Resour. Econ..

[33] Sanjarnia P., Picchio M.L., Polegre Solis A.N., Schuhladen K., Fliss P.M., Politakos N., Metterhausen L., Calderón M., Osorio-Blanco E.R. (2024). Bringing Innovative Wound Care Polymer Materials to the Market: Challenges, Developments, and New Trends. Adv. Drug Deliv. Rev..

[34] Joudeh N., Linke D. (2022). Nanoparticle Classification, Physicochemical Properties, Characterization, and Applications: A Comprehensive Review for Biologists. J. Nanobiotechnol..

[35] Zhang S., Jin L., Arshad M., Ullah A., Sharma H., Muresanu D., Sharma A. (2017). Renewable Biomaterials as Nanocarriers for Drug and Gene Delivery. Drug and Gene Delivery to the Central Nervous System for Neuroprotection.

[36] Sterne J.A., Hernán M.A., Reeves B.C., Savović J., Berkman N.D., Viswanathan M., Henry D., Altman D.G., Ansari M.T., Boutron I. (2016). ROBINS-I: A Tool for Assessing Risk of Bias in Non-Randomised Studies of Interventions. BMJ.

[37] Strotmann U., Thouand G., Pagga U., Gartiser S., Heipieper H.J. (2023). Toward the Future of OECD/ISO Biodegradability Testing—New Approaches and Developments. Appl. Microbiol. Biotechnol..

[38] Pacheco-Quito E.M., Ruiz-Caro R., Veiga M.D. (2020). Carrageenan: Drug Delivery Systems and Other Biomedical Applications. Mar. Drugs.

[39] Domingo-Echaburu S., Dávalos L.M., Orive G., Lertxundi U. (2021). Drug Pollution & Sustainable Development Goals. Sci. Total Environ..

[40] United Nations Health. United Nations Sustainable Development. https://www.un.org/sustainabledevelopment/health/.

[41] United Nations Environment Programme (2023). Emissions Gap Report. https://www.unep.org/resources/emissions-gap-report-2023.

[42] (2012). Nanotechnologies—Guidance on Physico-Chemical Characterization of Engineered Nanoscale Materials for Toxicologic Assessment.

[43] European Chemicals Agency (ECHA) (2016). Guidance on Information Requirements and Chemical Safety Assessment. Part E: Risk Characterisation.

