# Supplementary material for: Renewable Feedstock Nanocarriers for Drug Delivery: Evidence Mapping and Translational Readiness
Source: Pharmaceutics. 2026 Mar 25;18(4):407. doi: 10.3390/pharmaceutics18040407 (PMC13118287; doi:10.3390/pharmaceutics18040407)
Supplement: Supplementary file 1 [file pharmaceutics-18-00407-s001.zip › pharmaceutics-4194296-supplementary.pdf]

Supplementary Materials

Renewable Feedstock Nanocarriers for Drug Delivery: Evidence Mapping and Translational Readiness

Renato Sonchini Gonçalves

Department of Engineering and Exact Sciences, Setor Palotina, Federal University of Paraná (UFPR), Palotina 85950-000, PR, Brazil; renato.sonchini@ufpr.br; Tel.: +55-98-985-149-235

Table S1. Database-specific search strategy and retrieval log (PRISMA-adapted). Last search date: 11 February 2026.

Database	Date searched	Search fields	Search string (database-adapted syntax)	Filters/limits	Records retrieved (n)
Scopus	11 Feb 2026	Title/Abstract/Keywords	("renewable resources" OR "biomass-derived materials" OR biobased OR bioeconomy OR "green chemistry") AND (nanotechnology OR nanomaterials OR nanocarriers OR "drug delivery" OR nanomedicine) AND (sustainable OR "eco-friendly" OR biodegradable OR "low-toxicity") AND (pharmaceutical OR therapeutic OR biomedical) Database-specific syntax (field tags, phrase handling, and truncation) was applied to the core Boolean structure reported in the Methods.	No year restriction; English-language filtering not imposed a priori.	12,984

Web of Science Core Collection	11 Feb 2026	Topic (TS: title/abstract/keywords)	("renewable resources" OR "biomass-derived materials" OR biobased OR bioeconomy OR "green chemistry") AND (nanotechnology OR nanomaterials OR nanocarriers OR "drug delivery" OR nanomedicine) AND (sustainable OR "eco-friendly" OR biodegradable OR "low-toxicity") AND (pharmaceutical OR therapeutic OR biomedical) Database-specific syntax (field tags, phrase handling, and truncation) was applied to the core Boolean structure reported in the Methods.	No year restriction; all WoS document types initially retained.	6,531

ScienceDirect	11 Feb 2026	Title/Abstract/Keywords (platform search)	("renewable resources" OR "biomass-derived materials" OR biobased OR bioeconomy OR "green chemistry") AND (nanotechnology OR nanomaterials OR nanocarriers OR "drug delivery" OR nanomedicine) AND (sustainable OR "eco-friendly" OR biodegradable OR "low-toxicity") AND (pharmaceutical OR therapeutic OR biomedical) Database-specific syntax (field tags, phrase handling, and truncation) was applied to the core Boolean structure reported in the Methods.	No year restriction; initial broad search then refined by platform tools.	2,041

PubMed/MEDLINE	11 Feb 2026	All fields with MeSH where applicable	("renewable resources" OR "biomass-derived materials" OR biobased OR bioeconomy OR "green chemistry") AND (nanotechnology OR nanomaterials OR nanocarriers OR "drug delivery" OR nanomedicine) AND (sustainable OR "eco-friendly" OR biodegradable OR "low-toxicity") AND (pharmaceutical OR therapeutic OR biomedical) MeSH terms were used where applicable, combined with free-text keywords; query was adapted to PubMed/MEDLINE syntax.	No year restriction; PubMed filters not applied beyond query structure.	1,388

Notes: Records retrieved correspond to the initial database hits prior to reference management in Zotero and subsequent structured thematic refinement. Full database-specific strings (including exact field tags and truncation operators) should match the platform exports and are summarized here using the core Boolean structure reported in the manuscript.

Table S2. Operational reasons for non-inclusion in the core evidence-map set (n = 97) at the title/abstract screening stage (PRISMA-adapted).

**Operational reason for non-inclusion in the core set**	**n**
Background context (conceptual support; non-nano focus)	34

Background context (DDS/biomaterials; non-nano or macro-scale systems)	9
Ambiguous at title/abstract (probable non-nano; full-text needed to confirm eligibility for core set)	34
Excluded as out-of-scope (no clear pharmaceutical/therapeutic interface; e.g., energy/materials/adsorption/packaging-dominant)	11
Not retained in final core set after evidence-map confirmation (insufficient explicit renewability/green evidence and/or nano-platform/DDS linkage at abstract level)	9
**Total not included in core**	**97**

Note: Categories reflect operational triage labels used during title/abstract screening and subsequent cross-checking against the final core evidence-map dataset. Counts sum to 97.
